# From single pioneers to complex pro- and eukaryotic microbial networks in soils along a glacier forefield chronosequence in continental Antarctica

**DOI:** 10.3389/fmicb.2025.1576898

**Published:** 2025-05-21

**Authors:** Rahma Amen, Lars Ganzert, Thomas Friedl, Nataliya Rybalka, Dirk Wagner

**Affiliations:** ^1^GFZ Helmholtz Centre for Geosciences, Section Geomicrobiology, Potsdam, Germany; ^2^Department of Zoology, Faculty of Science, Aswan University, Aswan, Egypt; ^3^Experimental Phycology and Culture Collection of Algae, University of Göttingen, Göttingen, Germany; ^4^Institute of Geosciences, University of Potsdam, Potsdam, Germany

**Keywords:** Antarctica, glacier forefield soils, extracellular DNA, intracellular DNA, bacteria, Eukarya, algae, microbial diversity

## Abstract

**Introduction:**

In the extremely dry and oligotrophic soils of East Antarctica, where low temperatures and humidity result in minimal biological turnover rates, extracellular DNA (eDNA) can persist over extended timescales. Differentiating between sequences from living, potentially active cells (intracellular DNA, or iDNA) and those from ancient, non-living organisms (eDNA) is crucial for accurately assessing the current microbial community and understanding historical microbial dynamics.

**Methods:**

This study was conducted along a chronosequence in the Larsemann Hills, East Antarctica, where soil samples were collected from sites at varying distances from the glacier. By employing DNA separation methods, we distinguished iDNA, which represents living cells, from eDNA derived from dead organisms. High-throughput sequencing was used to characterize bacterial and eukaryotic communities across different successional stages.

**Results:**

The DNA separation approach revealed distinct bacterial and eukaryotic community structures along the glacier transect. Actinobacteria were consistently abundant across all sites, while other phyla such as Chloroflexi, Gemmatimonadetes, and Proteobacteria thrived in extreme, nutrient-poor environments. Early successional stages were characterized by the simultaneous colonization of green algae Trebouxiophyceae and cryophilic fungi, alongside nitrogen-fixing bacteria, which contributed to initial soil development. The study also identified three distinct modes of microbial distribution, reflecting varying degrees of activity and adaptability.

**Discussion:**

Our findings provide new insights into microbial dynamics in extreme habitats and propose new hypotheses for microbial colonization in newly exposed soils. Moreover, they contribute to the ongoing debate in microbial ecology regarding the viability of dormant or dead cells and emphasize the need for refining DNA-based methods and exploring functional pathways to deepen our understanding of microbial succession in polar regions.

## Introduction

Global climate change significantly impacts soil microbial communities in glacier forefields of Polar regions (Wynn-Williams, 1996; Barry, 2006; Lee et al., 2017). As glaciers retreat, previously ice-covered terrains form soil chronosequences characterized by their distance from the glacier terminus. Microbial communities in a chronosequence begin with specialized pioneers and gradually evolve into complex species interactions over time (Zumsteg et al., 2012; Bajerski and Wagner, 2013). Thus, glacier forefields in East Antarctica serve as an ideal model system for studying the development of microbial communities, tracing their progression from single pioneers to complex prokaryotic and eukaryotic assemblages (Czechowski et al., 2016; Kleinteich et al., 2017). These forefields are characterized by extreme conditions, including limited nutrient availability, the absence of mycorrhiza, low biological turnover due to cold temperatures and low humidity, and restricted water availability in a biologically accessible form. Furthermore, East Antarctica’s geographical isolation and minimal human influence make it an exceptional environment for discovering uniquely adapted species not found elsewhere (Obbels et al., 2016).

Antarctic soil microbial habitats remain underexplored, suggesting that much of their diversity is still unknown (Niederberger et al., 2015; Tahon et al., 2016). Nevertheless, recent studies, such as Lee et al. (2019), have analyzed large-scale patterns of microbial biodiversity and identifying key abiotic and biotic drivers of community structure (Lee et al., 2019). Most high-throughput sequencing studies have focused on bacterial communities in Antarctic soils (Tytgat et al., 2016; Meier et al., 2019; Krauze et al., 2021), while others have examined eukaryotic communities using amplicon-based paired-end sequencing on Illumina MiSeq (Czechowski et al., 2016; Crous et al., 2020; Rybalka et al., 2023). In the study by Czechowski et al. (2016), fungal phylotypes were the most widespread, followed by non-algal protists, while only three green algal species were detected, likely due to the absence of algal group-specific primers. However, a considerable diversity of eukaryotic algae has been recovered from fellfield soils in ice-free Maritime Antarctica (Rybalka et al., 2023). Molecular ecological studies of terrestrial Antarctic environments still lack species-level identification of eukaryotic algae. For example, low algal species diversity is often reported because algae are typically classified at low taxonomic resolution, such as the phylum or class level, e.g., Chlorophyta and Xanthophyceae (Magalhães et al., 2012). Despite their significant role in Antarctica’s terrestrial microbiota, the diversity and functional roles of eukaryotic algae in these environments remain poorly understood.

Concurrent investigations of bacterial and eukaryotic communities, alongside geochemical analyses of soil properties, are crucial for uncovering potential species interactions and cross-domain associations in glacier forefield soils. Simultaneous analysis of bacterial and eukaryotic communities remains rare, especially in Antarctica. Most studies have focused either on Bacteria alone (Teixeira et al., 2010; Bajerski and Wagner, 2013), Eukarya alone (Czechowski et al., 2016), or on Bacteria together with fungi or algae (Garrido-Benavent et al., 2020). An exception is a recent study that examined microbial succession along a glacier forefield in the Antarctic Peninsula, considering both bacterial and eukaryotic communities (Vimercati et al., 2022). Additionally, Lee et al. (2019) analyzed a large-scale dataset comprising microbial, fungal, and multicellular taxa across 500 sampling sites to provide further information about the complexity of Antarctic ecosystems. However, only a few studies have combined molecular and geochemical analyses to investigate the structure and development of bacterial communities in relation to soil properties in Antarctica (Ganzert et al., 2011; Bajerski and Wagner, 2013; Vimercati et al., 2022). Notably, Monteiro et al. (2022) linked changes in microbial community structure and diversity to spatial variations in geochemistry that drive microbial responses.

In the extremely dry and oligotrophic soils of East Antarctica, where low turnover rates result from cold temperatures and low humidity, extracellular DNA from non-living or non-metabolizing cells may be preserved from organisms that decomposed over timescales ranging from a few days to geological periods. Therefore, in such an extreme, low-biomass environment, it is crucial to differentiate between sequences derived from living, potentially active communities – i.e., intracellular DNA from intact cells – and those originating from relic organisms (Bartholomäus et al., 2024; Carini et al., 2017). Separating these DNA pools provides a more accurate representation of the living microbial community through the iDNA pool and, additionally, increases the amount of extractable DNA (Schulze-Makuch et al., 2018; Vuillemin et al., 2018; Medina Caro et al., 2023; Horstmann et al., 2024). Analyzing the eDNA pool, in turn, offers a unique opportunity to reconstruct ancient microbial populations and gain insights into microbial community changes over time.

We applied this innovative DNA separation method to investigate the simultaneous development of prokaryotic and eukaryotic communities in the glacier forefield of the Larsemann Hills, across sites at varying distances from the glacier front. By recovering intracellular DNA, we revealed a potentially viable microbial community in surface soils (0–30 cm), reflecting different times of exposure following glacier retreat, depending on the distance from the glacier front. Our approach aims to provide a more accurate depiction of the dynamics of living microbial communities in these extreme environments. We offer new insights into the viable fraction of microbial communities and the interactions between prokaryotic and eukaryotic microorganisms during soil development in the ice-free oases of East Antarctica.

## Materials and methods

### Site description and sampling

The sampling area is located in the ice-free area of the Larsemann Hills along the Ingrid Christensen Coast at Prydz Bay, East Antarctica (69°30’S, 76°20′E). The area has been deglaciated since 18–40 ka BP (Burgess et al., 1994; Hodgson et al., 2001; Kiernan et al., 2009). However, remnants of massive ice blocks can be found in some places, being no longer connected to the inland ice sheet. The climate in the region is continental with a marine influence leading to intensive physical weathering processes (Stüwe and Powell, 1989; Burgess et al., 1994). Air temperatures in the region are about −18°C to −29°C in Antarctic winter and around 0°C in summer. Precipitation usually occurs as snow with around 250 mm per year (Hodgson et al., 2001). Permafrost was detected in the soils, with an average active layer depth of 0.7 m in summer, depending on the site conditions (Vieira et al., 2010).

**Figure 1:**
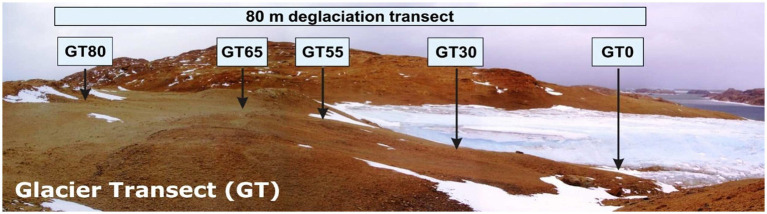
Picture showing the field site and the schematic position of the transect and sampling sites. GT, Glacier Transect, numbers specify the distance from the glacier in meter.

Soil samples were taken along a glacier forefield on Broknes Peninsula, which is one of the two main peninsulas of the Larsemann Hills, during the expedition ANT-XXIII/9 of the research vessel ‘Polarstern’ (Miller and Wagner, 2008). The sampled transect has been called ‘Glacier Transect - GT’ (S69°24.140^′^E76°20.178^′^ to S69°24.135^′^; E76°20.296^′^), spanning about 80 m distance (Figure 1). The surface along the transect was characterized by a stone or gravel pavement without any significant differences at the different study sites, except for the oldest site (GT80, see below), which was characterized by small spots (< 1 cm) of brown mosses. Due to limitations in accurate data on the age of the sites along such a short glacier forefield, we primarily selected the sampling locations according to visible changes. Even if the soils along the transect are predominantly skeletal and unstructured, in most cases without distinct classical genetic horizons (i.e., distinct layers in a soil profile that form as a result of soil-forming processes, e.g., weathering, organic matter accumulation), it was possible to differentiate the soils and select the specific study sites according to abrupt shifts in soil texture or soil color as well based on the relief in front of the glacier. Based on these criteria, five soil profiles were excavated along the transect, starting next to the glacier with GT0 (distance from glacier 0 m, youngest soil), continuing with GT30 (distance 30 m), GT55 (distance 55 m), GT65 (distance 65 m) and ending with GT80 (distance 80 m, oldest soil). Samples were taken from the soil profile after the profile wall was “cleaned” with a sterile brick trowel, and samples were then taken from each horizon. Depending on the depth of the soil, 2–3 horizons were sampled up to the bedrock to capture variations in microbial communities across the soil profile. The sampled horizons typically included the surface layer (0–10 cm), an intermediate layer (10–20 cm), and a deep layer (20–30 cm or until bedrock). For each layer, two replicates in the form of composite samples were taken over the entire horizon to consider the heterogeneity of the individual horizons. A total of 26 samples were taken from 13 soil layers, including two replicates each. Information on the samples and the texture of the soil are given in the Supplementary Table S1.

While microbial communities respond to environmental changes at a finer scale than macroscale soil descriptors, our sampling design aimed to capture broad shifts in soil properties that influence microbial succession over time. Additionally, parameters such as pH and soil moisture (Supplementary Table S1) were considered to account for environmental variability beyond visual soil characteristics, and the use of composite samples helped mitigate small-scale heterogeneity within each level.

For molecular biological analysis, the samples were collected in sterile 250 mL plastic containers (Nalgene). Samples for geochemical and geophysical analysis were stored in plastic bags. All samples were transported at −25°C on the research vessel ‘Polarstern’ from Prydz Bay (Antarctica) to Bremerhaven (Germany). According to the definition of Bockheim, and for simplicity, all materials in this study are referred to as ‘soil’ (Bockheim, 1982; Ugolini and Bockheim, 2008).

### Geochemical parameters

Conductivity and pH were measured in a soil extract (9 g soil in 45 mL MilliQ water) directly in the field. The soil slurry was filtered before the pH value measurement, whereas conductivity was measured directly in the extract. Moisture content was determined by freeze-drying the soil by weighing the sample before and after. Total carbon and nitrogen contents were measured with an automatic element analyzer (Elementar Vario EL III), as described in detail elsewhere (Wagner et al., 2007). For water extractable ion analyses, a soil slurry (9 g dry soil + 25 mL Milli Q water) was mixed in an overhead shaker for 90 min and centrifuged at 3500 g for 20 min. Anions were measured by ion chromatography (Dionex-DX320). Grain size distribution was determined as described by Biskaborn et al. (2012) and measured in a laser particle analyzer (Coulter LS 200).

### Separation and extraction of extracellular and intracellular DNA

This study introduces a modified protocol for the separation and extraction of extracellular DNA (eDNA) and intracellular DNA (iDNA) from soil samples, particularly those with low organic matter content. This approach addresses limitations encountered with existing methods, specifically targeting the challenge of low DNA yield in such environments. To distinguish between extracellular DNA (eDNA) and the DNA of intact cells, we adapted and optimized the protocol originally described by Alawi et al. (2014), with further modifications based on Medina Caro et al. (2023). Due to the anticipated low carbon concentrations, we omitted the use of polyvinylpolypyrrolidone, which can hinder downstream analyses by removing humic acids but also potentially co-precipitating target DNA. To compensate for this omission and ensure efficient cell lysis, we increased the sample amount to 3 g. Additionally, we proportionally increased the volume of the sodium phosphate buffer used in the washing step to ensure thorough removal of humic acids and other potential inhibitors. Detached cells were then collected by filtration onto 0.22 μm Sterivex filters (Millipore) instead of centrifugation. To achieve efficient binding of eDNA, we employed a modified strategy compared to the original protocol. We added twice the recommended volume of 6 M sterile-filtered (0.22 μm) guanidinium hydrochloride (GuaHCl) and 60 μL silica suspension. This enhanced the capture and recovery of eDNA from the environmental matrix. iDNA was extracted from Sterivex filters using a well-established CTAB-phenol-chloroform-isoamylalcohol bead-beating protocol (Perkins et al., 2019). DNA concentrations were measured using the Qubit system with the dsDNA HS Assay Kit (Invitrogen); however, they were very low (*<* 1 ng μL^−1^) for most of the samples.

To ensure the reliability of our results, procedural controls were implemented at multiple stages, including DNA extraction, library preparation, sequencing, and qPCR. Negative controls were included during DNA extraction and library preparation to monitor potential contamination. These controls were sequenced alongside the samples, and any contaminating sequences that appeared in comparable or greater abundance in at least one of the negative controls were carefully removed from the dataset. Additionally, extraction replicates were assessed for consistency, and samples for which two or more replicates showed contamination or low sequencing read counts were excluded from downstream analyses. For qPCR, both extraction negative controls and non-template controls were included to detect and eliminate potential contamination during amplification.

### Polymerase chain reaction

For amplification of the V3-V4 hypervariable region of the bacterial 16S rRNA gene the primer set Bac341F (5’-CCTACGGGNGGCWGCAG-3′) and Bac805R (5’GACTACHVGGGTATCT AATCC-3′) (Herlemann et al., 2011) was used. PCR conditions for Bacteria were as follows: 95°C for 5 min, 30 cycles of 95°C for 30 s, 55°C for 30 s and 72°C for 45 s, with a final elongation step at 72°C for 10 min. Eukaryotic 18S rRNA gene fragments of the V4 hypervariable region were amplified with the primer pair TAReuk454FWD1 (5’-CCAGCASCYGCGGTAATTCC-3′) and TAReukREV3 (5′- ACTTTCGTTCTTGATYRA-3′) (Stoeck et al., 2010). PCR conditions for Eukarya were 95°C for 5 min, 35 cycles of 95°C for 30 s, 48°C for 45 s and 72°C for 30 s, with a final step at 72°C for 10 s. All primers were labeled with an 8–10 nt barcode. PCR products were visually checked on a 1.2% agarose gel. Their concentrations were measured using a Qubit system with the dsDNA HS Assay Kit (Invitrogen) before equally pooling the PCR products for library preparation.

### Sequencing and bioinformatics

Library preparation (2 × 300 bp V3 kit) and sequencing on a MiSeq Illumina platform was done by Eurofins Genomics (Germany). Raw paired reads were sorted and demultiplexed using a combination of Mothur v1.37.6 (Schloss et al., 2009), Qiime v1.9 (Caporaso et al., 2010), and bash commands. After demultiplexing, DADA2 v1.8 (Callahan et al., 2016) was used for sequence quality check, error correction, and chimera detection for bacterial 16S rRNA gene and eukaryotic 18S rRNA gene amplicons. Amplicon sequence variants (ASVs) were classified using a naive Bayesian classifier as implemented in DADA2 (Wang et al., 2007), with Silva v138 as a reference database (Quast et al., 2012). The results for Eukarya were refined through a query of all eukaryotic ASV representatives against the whole GenBank Nucleotide database (NCBI-GenBank Flat File Release 259.0 of December 15, 2023), and the taxonomic labels determined comparing the query-reference similarities for reference sequences using the consensus approach as in Rybalka et al. (2023). For the Eukarya, names of taxonomic groups were manually adjusted to follow recent Eukarya taxonomies (Adl et al., 2019; Büdel et al., 2024). Global singleton sequences and sequences classified as Archaea, mitochondria, chloroplasts and vertebrate were excluded from the dataset.

We implemented a quality filter on the database of technical replicates for samples of both Bacteria and Eukarya. This filter employed a comprehensive analysis considering multiple factors: Non-Metric Multidimensional Scaling (NMDS) for data structure visualization, Species Richness to assess overall diversity, Pielou’s Evenness to evaluate community composition balance, visual inspection of the data, and DNA concentration assessment. Any technical replicate exhibiting significant discrepancies compared to its counterparts was removed. This resulted in the exclusion of one replicate from five Bacteria samples and three complete samples for the Eukarya data (GT65 20–30 cm both eDNA and iDNA, GT65 10–20 cm iDNA) due to insufficient sequence counts. The final ASV table was then used for visualization and statistical analysis. All sequence reads are available in the Sequence Read Archive (SRA) under the BioProject number PRJNA685954.

### Data analyses and visualization

Statistical analyses were done in R 3.6.1 (R Core Team, 2019) or PAST3.25 (Hammer et al., 2001). ASV tables were subsampled to 26,000 reads per sample for Bacteria and to 3,200 reads per sample for Eukarya, respectively, using the rtk package (Saary et al., 2017) to normalize sequencing depth and enable robust comparisons of microbial diversity across samples. While rarefaction may result in the loss of some valid data, this approach is widely used to address uneven sequencing depth and minimize bias in diversity analyses. The chosen thresholds were based on the distribution of read counts to ensure that the majority of samples were retained while maintaining sufficient sequencing depth for reliable diversity estimates. Sample dissimilarities were visualized with PAST3.25 using non-metric multidimensional scaling (NMDS) for Bacteria and canonical correspondence analysis (CCA) for Eukarya based on a Hellinger-transformed ASV matrix and Bray-Curtis distance. CCA was chosen for the visualization of the eukaryotic communities because of a stress value well above 0.2 in the NMDS. Alpha diversity values for species richness and Pielou’s Evenness were calculated and plotted for both DNA pools using PAST3.25. The differences in alpha diversity between the two DNA pools were tested using *t*-test for statistical significance. Venn diagrams were drawn using an online tool^1^ and customized with Corel Draw.

ASV tables were subsampled to 26,000 reads per sample for Bacteria and to 3,200 reads per sample for Eukarya to normalize sequencing depth and enable robust comparisons of microbial diversity across samples. While rarefaction may result in the loss of some valid data, this approach is widely used to address uneven sequencing depth and minimize bias in diversity analyses (Weiss et al., 2017; McMurdie and Holmes, 2014). The chosen thresholds were based on the distribution of read counts, ensuring that the majority of samples were retained while maintaining sufficient sequencing depth for reliable diversity estimates.

Weighted Correlation Network Analysis (WGCNA) was used to identify modules, that is clusters of highly correlated bacterial and eukaryotic ASVs, and to relate these modules to environmental traits (e.g., water content, total carbon, total nitrogen, pH, electrical conductivity, anion concentrations, and soil texture) using the *wgcna* package (Langfelder and Horvath, 2008). For that, only the iDNA pool was used to avoid potential misleading correlations between past and present community members. The underlying dataset was based on combined bacterial and eukaryotic ASVs that appeared with relative abundances of 0.5% across the whole dataset to reduce network complexity. Cytoscape was used for visualizing the correlations between modules and environmental parameters (Shannon et al., 2003). For the corresponding co-occurrence network pair-wise Pearson correlations between the ASVs were calculated using the *rcorr* function in the *Hmisc* package (Harrell and Harrell, 2019) and only strong correlations with *R* > 0.8 and *p* < 0.01 were kept for visualization with the *igraph* package (Csardi and Nepusz, 2006). Topological network properties such as modularity index (indicating how modular a network is; Newman, 2006), clustering coefficient (the degree nodes cluster together) and diameter (longest distance in the network) were calculated using the *igraph* package (Csardi and Nepusz, 2006).

Indicator Species Analysis (ISA) was done using the *multipatt* function in the package *indicspecies* (De Cáceres and Legendre, 2009), using the same dataset as for WGCNA, with samples grouped by site. Specialist species were defined as being an indicator ASV in one of the five sites while generalist species were defined as being an indicator ASV appearing in at least four sites. Additionally, for the phototrophic Eukarya (green algae and bryophytes) we included only the surface samples to define generalist and specialist phototrophic Eukarya. Again, phototrophic ASVs with a minimum relative abundance of 0.5% across all samples were chosen and considered specialist ASVs when occurring in only one site with at least 0.5% relative abundance.

## Results

### Diversity of the microbial communities along the GT chronosequence

The sequencing of the microbial community revealed 9,793,579 and 3,359,645 raw reads belonging to Bacteria and Eukarya, respectively. After quality check we obtained 5,386,403 for Bacteria, with an average of 103,585 reads per sample (ranging from 15,305 to 239,271) and 2,642,463 reads for Eukarya, with an average of 53,928 reads per sample (ranging from 4 to 200,863).

**Figure 2:**
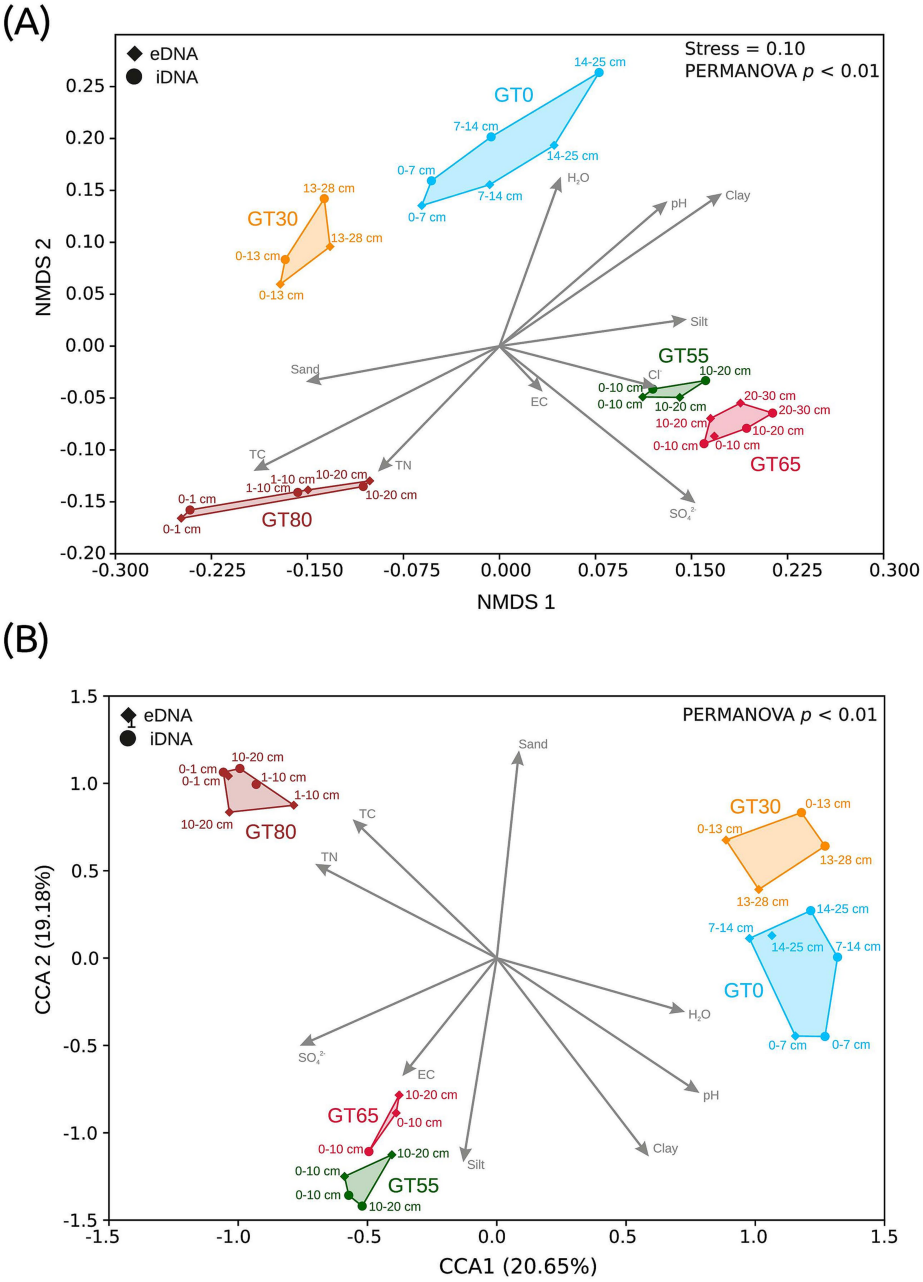
Ordination plots of the microbial communities for the separated eDNA and iDNA pools, based on Bray-Curtis dissimilarities. **(A)** Non-metric multidimensional scaling (NMDS) for Bacteria, **(B)** canonical correspondence analysis (CCA) for Eukarya. Note: the vectors displaying the environmental variables were upscaled in the CCA by a factor of 1.5 for better visualization. GT, Glacier Transect, following numbers display the distance in meters from the glacier.

A significant segregation in the soil microbial communities along the glacial transect (Bacteria: *p* < 0.01; Eukarya: *p* < 0.01) was evidenced in multivariate ordinations (NMDS for Bacteria and CCA for Eukarya, based on Bray-Curtis dissimilarities; Figure 2). The samples of each study site along the glacier transect (GT) were clustered together, without direct overlap with other clusters. These clusters can be further separated by the site age, sample depths, and DNA pools (eDNA and iDNA). The young sites close to the glacier front (GT0 and GT30), the middle sites farther from the glacier front (GT55 and GT65), and the oldest site farthest from the glacier front (GT80) could be distinguished from each other. The depths of the samples were separated from each other in most cases, especially for bacteria (Figure 2A). Also, the microbial community based on the eDNA and iDNA pools for both bacteria (Figure 2A) and microbial eukaryotes (Figure 2B) were aggregated, but still associated in the same cluster. For both the bacterial and eukaryotic communities, the separation of the sites correlated with important environmental parameters, i.e., water content, anions, total carbon, total nitrogen, and pH value. For instance, the closest site to the glacier (GT0) was positively correlated with the water content. The sites GT55 and GT65 were correlated mainly with anions (sulfate, chloride) and silt, while the site GT80 was correlated with total carbon (TC), total nitrogen (TN), and a lower pH value. The geochemical soil properties along the chronosequence are given in the Supplementary Table S2.

### Species diversity and evenness

**Figure 3:**
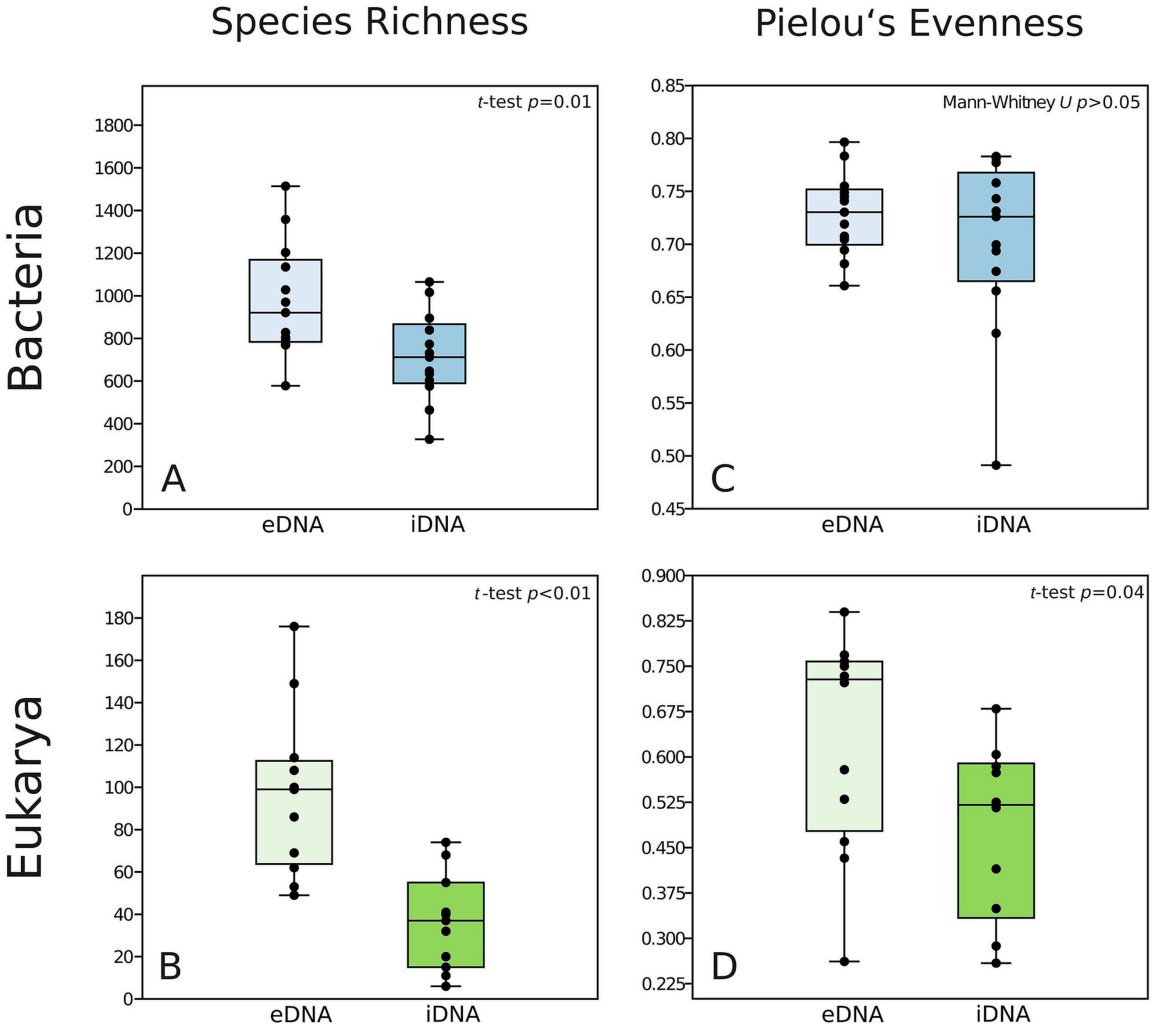
Boxplots of the differences in alpha diversity between the separated eDNA and iDNA pools showing Species Richness **(A,B)** and Pielou’s Evenness **(C,D)** for Bacteria (blue) and Eukarya (green).

Alpha diversity indicator in terms of ASV richness showed that there were significant differences between eDNA and iDNA pools for both Bacteria (Figure 3A; Supplementary Figure S1A; *t*-test *p* = 0.01) and Eukarya (Figure 3B; Supplementary Figure S1C; *t*-test *p* < 0.01) communities. For Bacteria, the species richness was about 10 times higher (578–1,514 ASVs in the eDNA and 327–1,065 ASVs in the iDNA, Figure 3A; Supplementary Figure S1A) than for the Eukarya (49–176 ASVs in the eDNA and 6–74 ASVs in the iDNA, Figure 3B; Supplementary Figure S1C). Species richness closest to the glacier front was in the same range or even higher at the sites farther from the glacier (Supplementary Figures S1A,C).

With sample depth, the richness of bacteria and eukarya species in the iDNA pool decreased (Supplementary Figure S1). However, for the eDNA pool, bacterial richness showed a slight increase with depth at GT30, GT55, and GT65, and a clear increase at the site GT80, likely due to the accumulation of extracellular DNA from past microbial communities in deeper soil layers. There was no clear trend for Eukarya at the same site.

Pielou’s evenness index indicated that evenness values and variation differed between the separated DNA pools and between Bacteria and Eukarya (Figures 3A,D). In Bacteria, differences in the evenness of both separated DNA pools were insignificant (Figure 3C; Mann–Whitney *U*-test *p* > 0.05), although variation in evenness was lower in the eDNA than in the iDNA. However, in Eukarya, the evenness of the iDNA pool was significantly lower than that of the eDNA pool (*t*-test *p* = 0.04). Evenness variation was generally lower for Bacteria than for Eukarya (Figures 3C,D). With the latter, there was an evenness increase in both DNA pools with distance from the glacier front (Supplementary Figures S1B,D). For the bacterial eDNA pool, the evenness values were within the same range across all five study sites of the transect.

### Proportions of eDNA/iDNA along the glacier transect

**Figure 4:**
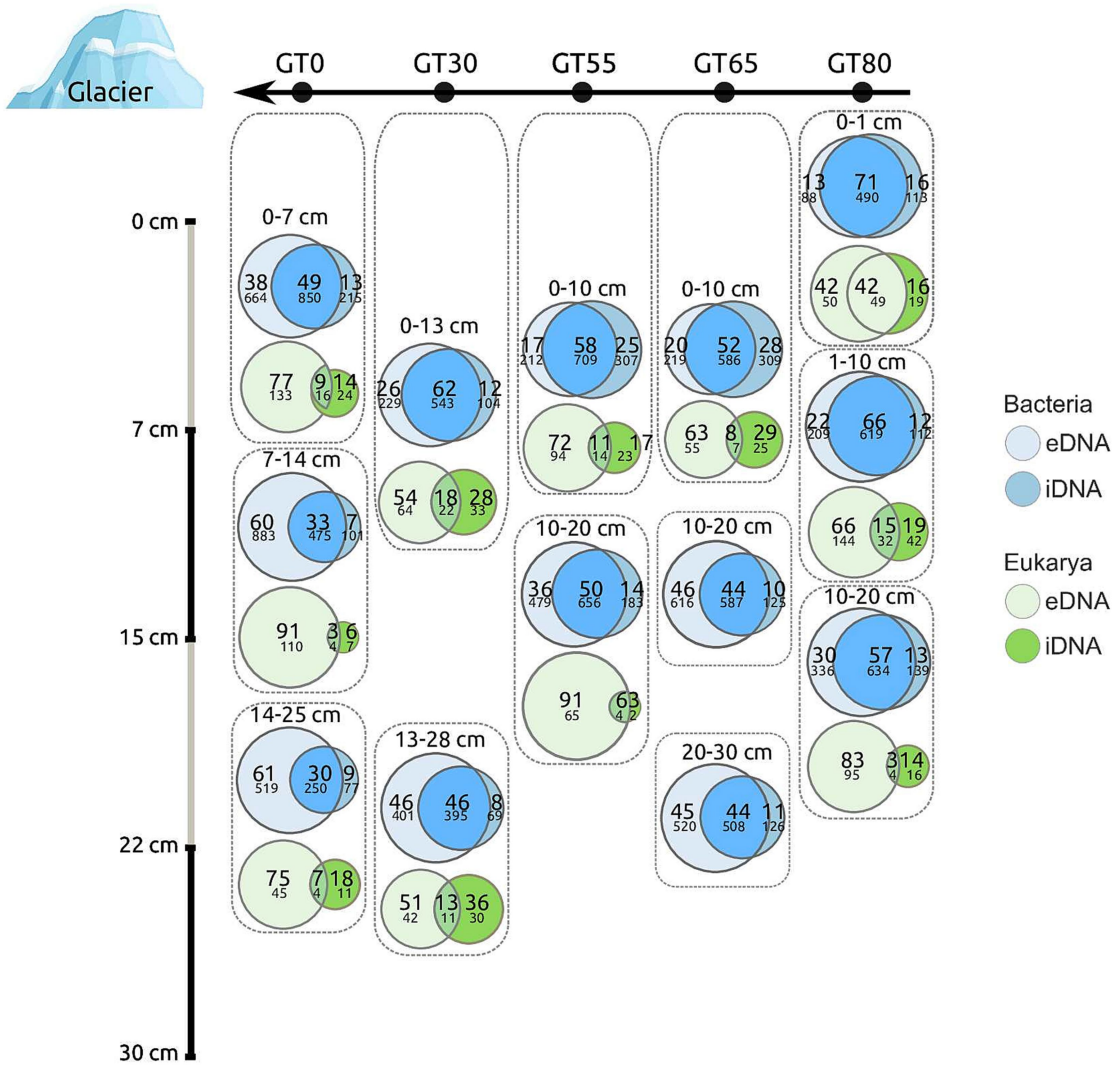
Venn diagrams showing the proportions of Bacteria (blue) and Eukarya (green) ASVs that were either unique to the eDNA or iDNA pool or shared by both DNA pools. Each Venn diagram represents a sample at the different sites across the investigated soil chronosequence, with their corresponding depth in centimeters. Large (upper) numbers in the Venn diagram compartments display the relative proportion of ASV numbers [in %], and small (lower) numbers are the absolute ASV numbers per sample.

Venn diagrams, shown in Figure 4, visualized three fractions of ASVs, i.e., those unique to either the eDNA or the iDNA pool, and those shared by both DNA pools. The diagrams show that the proportions of both separated DNA pools varied along the glacier transect and with sample depth (Figure 4). Overall, the absolute ASV numbers of each fraction were considerably higher for Bacteria than for Eukarya, which corresponded to the observed species richness (Figures 3A,B). In the Eukarya, the unique eDNA fraction dominated, i.e., it mostly comprised *>* 50% of the ASVs, ranging from 42% at site GT80, top layer, to 91% at site GT0, middle layer (Figure 4). The unique iDNA and shared fractions from both pools were considerably smaller compared to the unique eDNA fraction. In Bacteria, the fraction shared by both separated DNA pools dominated, i.e., it ranged from 30% at site GT0, lower layer, to 71% at site GT80, top layer (Figure 4). The unique eDNA fraction in both Bacteria and Eukarya increased from the top to the middle or lower layers of the soil. Another increase in the shared fractions of Bacteria and Eukarya was observed with increasing distance from the glacier front, i.e., from site GT0 to site GT80.

### Bacterial community structure

In both separated DNA pools, a total of 24 bacterial phyla were recovered, dominated by Actinobacteria, Chloroflexi, Gemmatimonaetes, Patescibacteria, Protobacteria, Verrucomicrobia, and WPS-2 (proposed as *Candidatus* phylum Eremiobacterota). Some dominating bacterial phyla exhibited different abundances between the DNA pools and among the GT sites. For example, the phylum Actinobacteria exhibited high abundances at all sites and depths, while the Gemmatimonadetes and Patescibacteria showed high abundances close to the glacier front (sites GT0 and GT30) and at deep soil levels in both DNA pools. The Rokubacteria and WPS-2 exhibited the highest abundance in the iDNA pool of the site GT30. The photoautotroph Cyanobacteria had their highest abundances in both DNA pools in the top layer of the sites closest to the glacier front, GT0 (Supplementary Figure S2).

**Figure 5:**
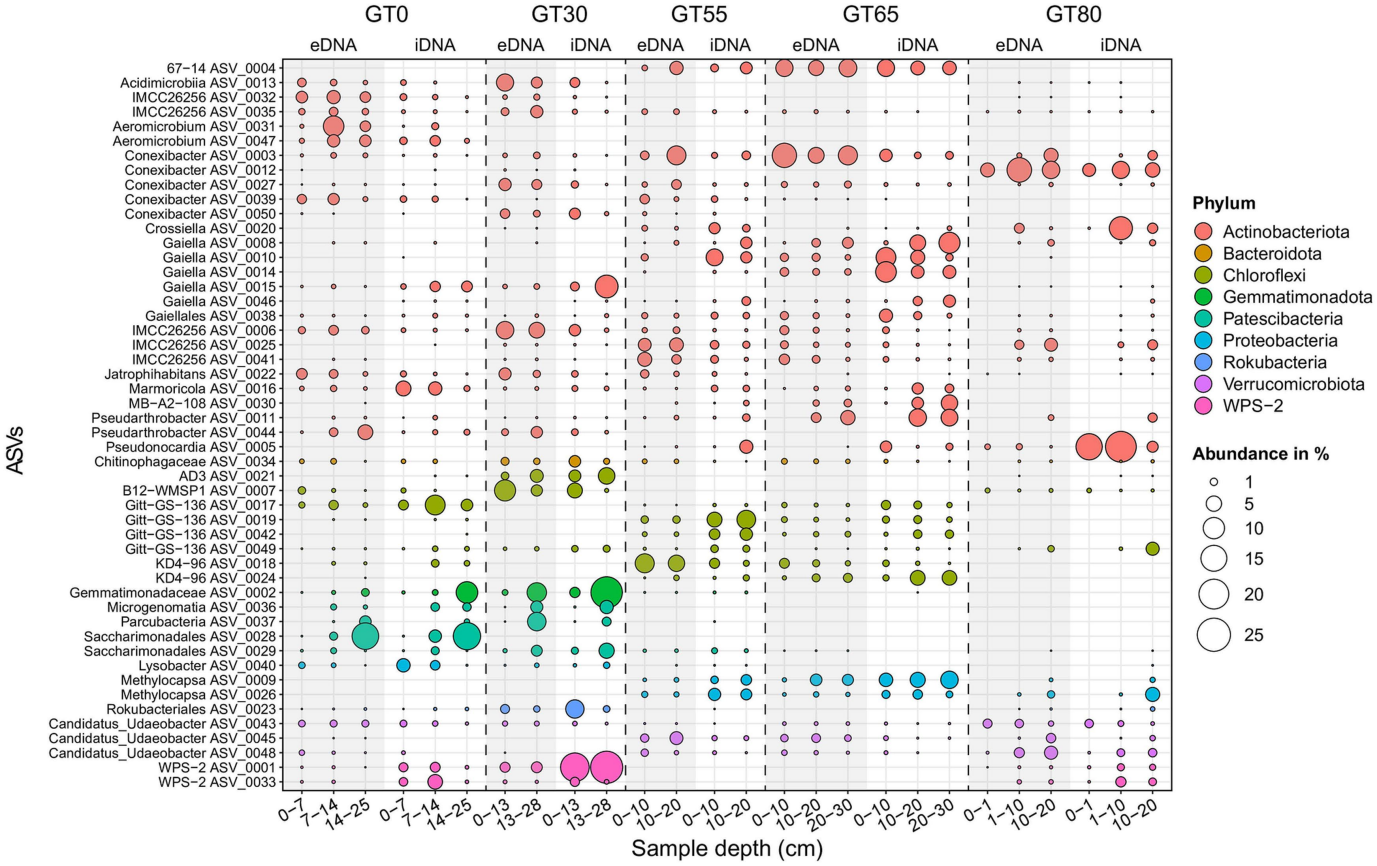
Bubble plot showing the distribution and structure of the 50 most abundant bacterial ASVs in both separated DNA pools (eDNA, gray background; iDNA, white background). Each filled circle represents the occurrence of a certain ASV with its size corresponding to their relative abundance. ASVs are sorted by phylum and characterized by their genus name when possible, otherwise the next highest classification level was chosen. A similar figure at the phylum level is given in the Supplementary Figure S2.

At high taxonomic resolution, most of the top 50 bacterial ASVs belonged to the phyla Actinobacteria (27 ASVs) and Chloroflexi (8 ASVs). Most Bacteria top 50 ASVs were restricted to certain GT sites (Figure 5). For example, *Acidimicrobiia* ASV 0013 and *Gaiella* ASV 0015 had considerable abundances only at the two sites close to the glacier front, GT0 and GT30 (Figure 5). *Aeromicrobium* ASV 0031, ASV 0047, and ASV 0028 (Patescibacteria, *Saccharimonadales*) appeared to be restricted to the site GT0. The ASV 0004 (Actinobacteria, group 67–14) was detected only at the middle sites of the chronosequence (GT55 and GT65). The ASV 0009 and ASV 0026 (both Proteobacteria, *Methylocapsa*) were absent at GT0 and GT30 but present at the other sites (Figure 5).

Depth was another factor influencing bacterial ASV relative abundances (Figure 5). For example, Saccharimonadales ASV 0028 (site GT0), Chloroflexi ASV 0021 (site GT30), and Gemmatimonadetes ASV 0002 (sites GT0 and GT30) showed an increase in relative abundance with depth. *Lysobacter* (Proteobacteria) ASV 0040 (site GT0) and Chloroflexi ASV 0007 (sites GT0 and GT30) showed a decrease in relative abundance.

Indicator species analysis (ISA) for the iDNA pool, using a 0.5% relative abundance threshold, revealed a total of 102 bacterial ASVs as significant indicator species (specialists) for a single site (see Supplementary Figure S4; Supplementary Table S3). Most indicator species belonged to the phyla Actinobacteria (35 ASVs) and Chloroflexi (21 ASVs). ASV 0006 (Actinobacteria, IMCC26256), ASV 0035 (Actinobacteria, Acidimicrobiia), and ASV 0043 (Verrucomicrobia, Candidatus Udaeobacter) were distributed almost evenly across all sites (Figure 5). ISA showed only two ASVs being indicator species for all five sites (generalists), i.e., the ASV 0035 (Actinobacteria, *Acidimicrobiia*) and ASV 0136 (FBP) (Supplementary Figure S4). The number of bacterial specialist species for two or more sites decreased from 117 (2 sites) to 15 ASVs (4 sites). Site GT80 (farthest from the glacier) exhibited the largest number of bacterial specialists ASVs (Supplementary Figure S4), which were unique (with a cutoff of 0.5% relative to the abundance) at that site.

### Eukaryotic community structure

**Figure 6:**
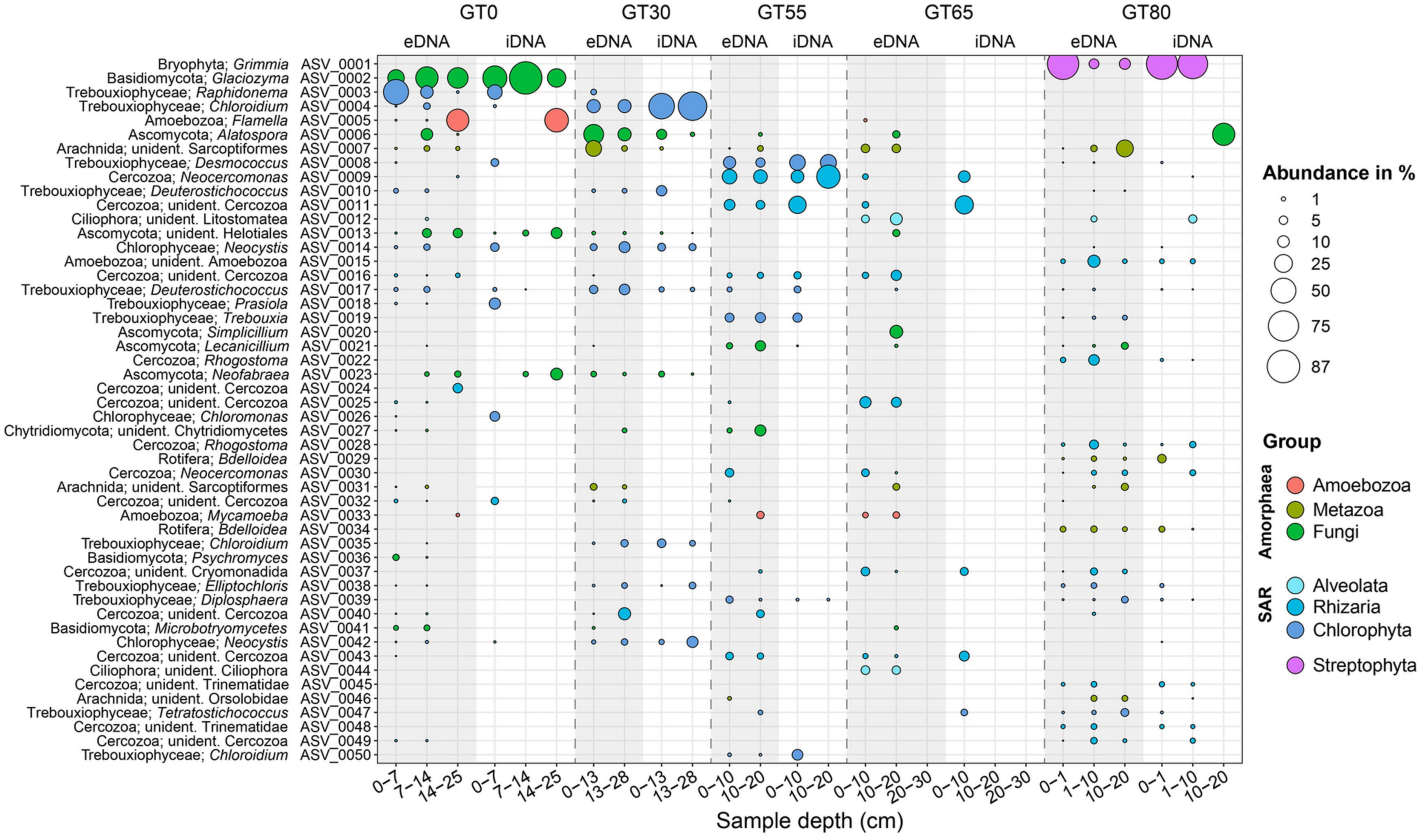
Bubble plot showing the distribution and relative abundances of the 50 most abundant eukaryotic ASVs in both separated DNA pools (eDNA, gray background; iDNA, white background). Each filled circle represents the occurrence of a certain ASV with its size corresponding to their relative abundance. ASVs are sorted by division and characterized by their genus name when possible, otherwise the next available classification level was chosen. NA - no data available. A similar figure at the phylum level is given in the Supplementary Figure S3.

Among the Eukaryote ASVs with the 50 highest read counts, the autotrophic green algae (i.e., Trebouxiophyceae 24%, and Chlorophyceae, 6%) and the heterotrophic Cercozoa were equally the most dominant groups, i.e., each formed 30% of the top 50 ASVs (Figure 6). They were followed by fungi (Ascomycota, 10%, Basidiomycota yeasts, 6%, and Chytridiomycota, 2%) and other heterotrophic life forms, i.e., small Metazoa, mites (Arachnida, 6%), Rotifera (4%), as well as Amoebozoa (6%), and unicellular ciliates (Ciliophora, 4%). A single ASV represented the bryophyte *Grimmia* which included the most read counts of all Eukaryota ASVs (Figure 6).

For both ends of the glacier transect, sites GT0 and GT80, no more than 13 top ASVs were shared by both sites. About half of them (6) represented autotrophic green algae. All other top ASVs were recovered from only one of the ends of the transect or one or more sites in between. Both ends of the transect comprised about the same number of top ASVs (32 at GT0 and 30 at GT80). Among the six top ASVs exclusively recovered from close to the glacier front (GT0), four were typical green snow algae (*Raphidonema* ASV_0003, *Chloromonas* ASV_0026) or psychrophilic fungi (*Glaciozyma* ASV_0002 and *Psychromyces* ASV_0036). Those had high abundancies of 25% and more. *Glaciozyma* ASV_0002 was even revealed as an indicator species for site GT0. The bryophyte *Grimmia* (ASV_0001), the cercozoan *Rhogostoma* (ASV_0022, ASV_0028), and unidentified Amoeboza ASV_0015 were recovered only from the other end of the transect, site GT80. The ASVs of the green alga *Chloroidium* were supported as specialists for a single site by ISA, i.e., two for site GT30 (ASV_0035 and ASV_0118; 0.5% relative abundance cutoff; Supplementary Table S4). A third (ASV_0050) was recovered exclusively from site GT55. In contrast, ISA supported the green alga *Desmococcus* ASV_0008 as a generalist species found at the three sites GT0, GT55, and GT80 (Figure 6; Supplementary Table S4).

Only six top ASVs were recovered exclusively from the (ancient) eDNA fraction, all others from both DNA fractions, and no ASV was found only in the iDNA. They represented only heterotrophs (mostly Cercozoa) and had abundancies below 10%. Remarkably, in the iDNA fraction, several green algae were found in depths up to 28 cm below surface, i.e., *Chloroidium, Deuterostichococcus, Elliptochloris* (all Trebouxiophyceae), and *Neocystis* (Chlorophyceae).

Additional taxonomic groups of eukaryotes were revealed when considering also those ASVs of lower relative abundances (Supplementary Figure S3). Out of the autotrophic algae the Xanthophyceae (Stramenopiles) were relevead in the eDNA and iDNA pools while the Chrysophyceae/Synurophyceae (Stramenopiles), the Ulvophyceae (Chlorophyta), and Klebsormidiophyceae (Streptophyta) were recovered only from the eDNA and mostly in low abundances. More eukaryotes found only in the eDNA fraction were from fungi (Saccharomycetales, Agaricomycetes), lineages of Amoebozoa and Obazoa (Supplementary Figure S3). Smaller Metazoa (e.g., Eutardigrada, Nematoda) and an increased diversity of Ciliophora were recovered from both DNA fractions, but mostly from sites GT65 and GT80 far from the glacier.

### Co-occurrence network analysis

**Figure 7:**
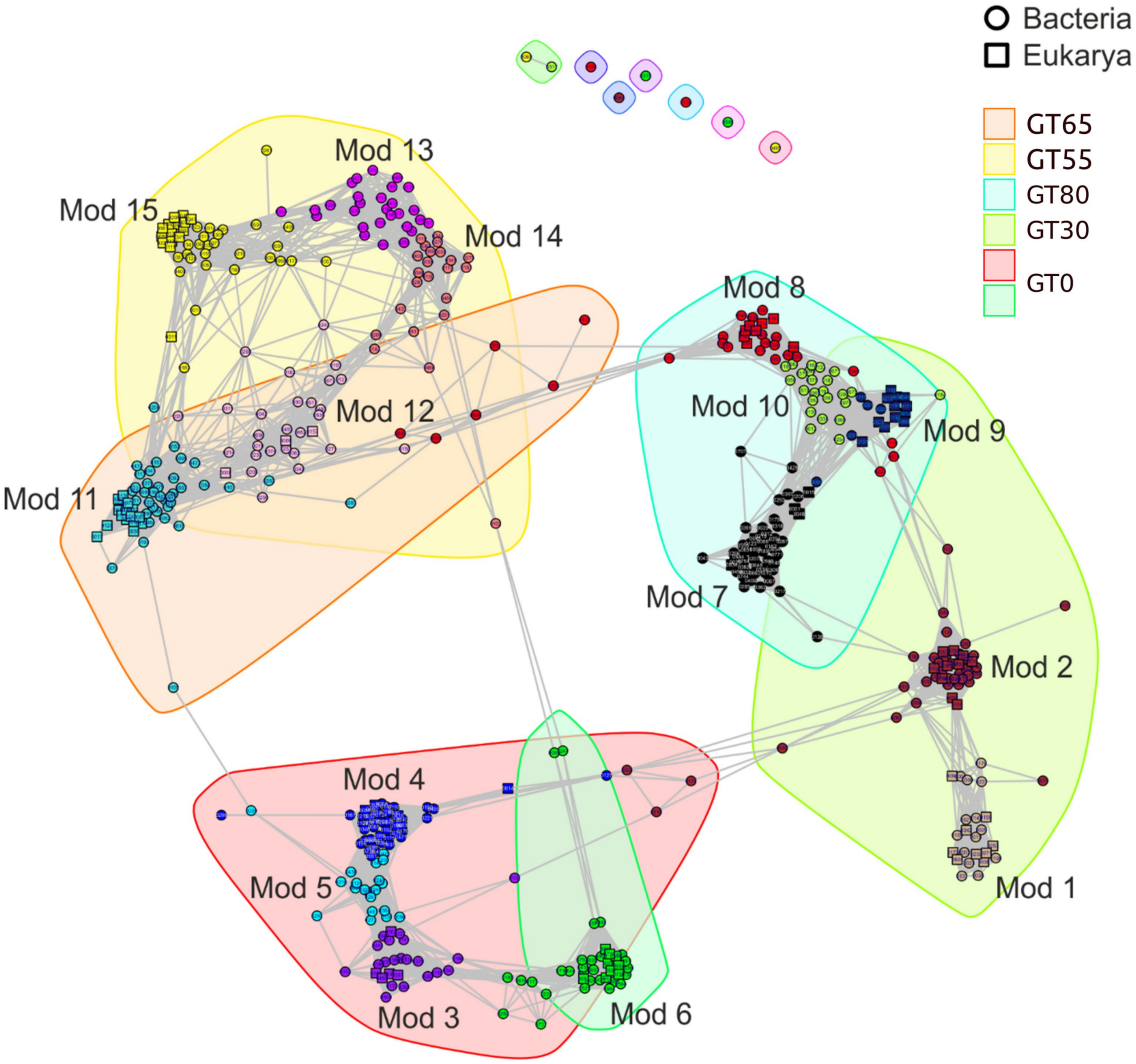
Co-occurrence network of 403 Bacteria and 102 Eukarya ASVs, respectively. ASVs are colored according to their module membership. Only strong positive correlations (*r* > 0.8, *p* < 0.01) are shown to decrease complexity. Mod, module.

The Weighted Correlation Network Analysis (WGCNA) was performed on a dataset of 505 ASVs, including 403 bacterial and 102 eukaryotic ASVs. The analysis resulted in a network of 505 nodes and 7,278 edges (Figure 7; Supplementary Table S5). The network displayed a clustering coefficient of 0.82, indicating a high level of interconnectedness among the nodes. The short path length was found to be 5.1, indicating a relatively small number of steps required to traverse the network. The modularity score of 0.83 suggested the presence of distinct, densely connected groups or modules within the network (Newman, 2006). The network diameter was 14, representing the largest number of steps required to traverse the network from one node to another. The graph density was calculated to be 0.06, indicating a relatively high level of interconnectedness in the network (Newman, 2006; Supplementary Table S5).

The network analysis revealed a higher node connectivity for the Bacteria ASVs (on average > 11 edges per node) compared to the Eukraya ASVs (on average > four edges per node). The interactions between the Bacteria and Eukarya were represented in the network by 28% of its edges (four edges per node). Node connectivity and the number of interactions for Bacteria and Eukarya were the highest at both ends of the chronosequence, i.e., the sites GT0 and GT80 (Supplementary Table S5).

The network analysis identified the keystone taxa, i.e., those with the highest degree centrality (the number of connections that a node has in a network) and greatest betweenness centrality (the extent to which a node in a network lies on the shortest path between other nodes, Newman, 2006). The Bacteria keystone taxa were mainly represented by Actinobacteria, Chloroflexi, Proteobacteria, and Gemmatimonadetes, while those of the Eukarya belonged to the Chlorophyta, Cercozoa, and Fungi. These taxa had the largest number of degrees, indicating that they were the most abundant and connected in the community and played a crucial role in the assembly of the bacterial communities. The greatest betweenness centrality was also observed in these taxa, demonstrating that they were positioned between other taxa and acted as connecting elements in the assembly of bacterial and eukaryotic communities. The keystone taxa in both the bacterial and eukaryotic communities were mainly found in sites GT0 (highest degree) and GT80 (highest betweenness centrality), see Supplementary Figure S5 for more details.

**Figure 8:**
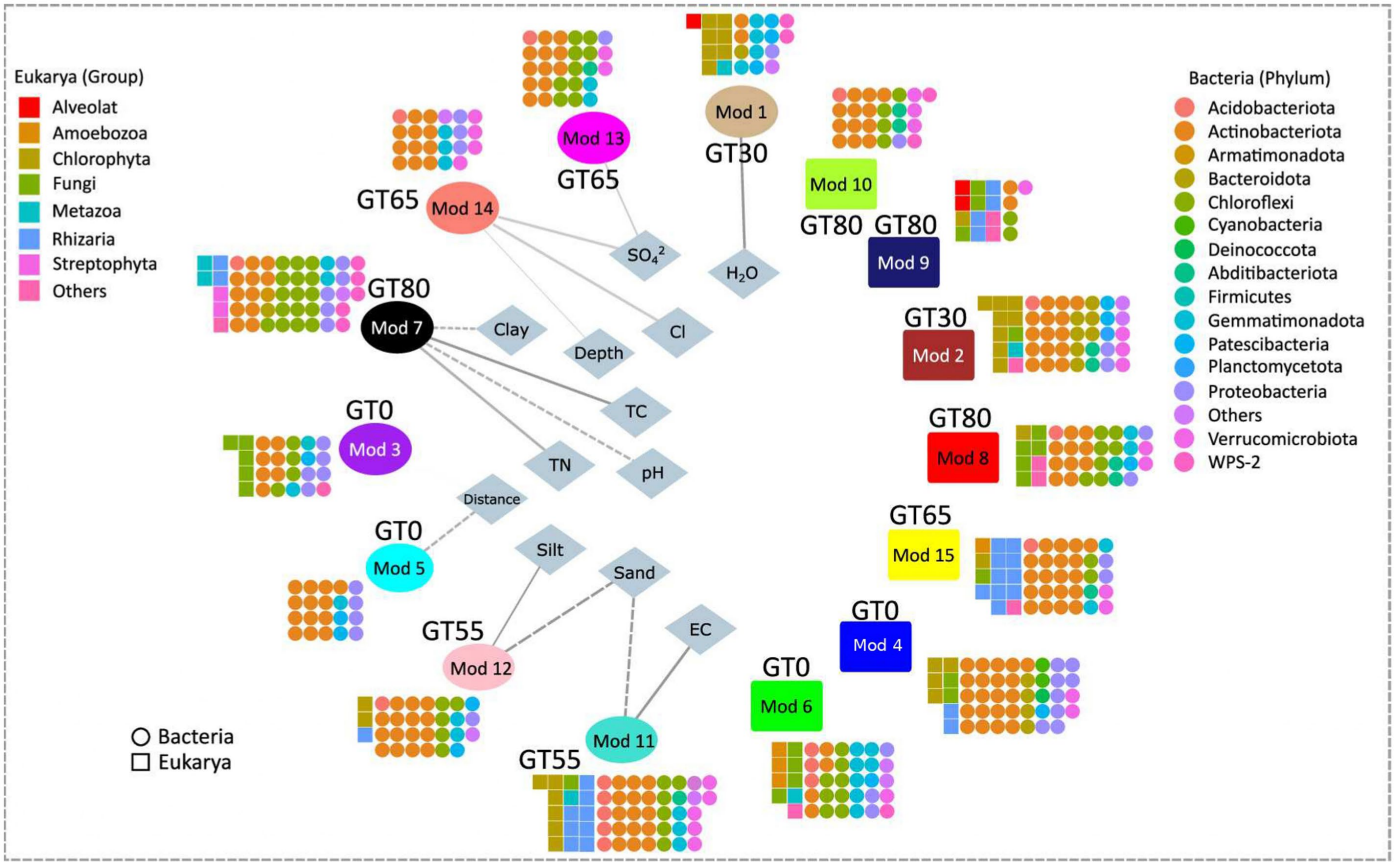
Modules of Bacteria and Eukarya co-occurring in the Glacier Transect (GT) and their correlation with different environmental parameters. Bacterial ASVs are shown in circles (colored according to phylum level) and eukaryotic ASVs as squares (colored according to taxonomic groups). Modules that correlated significantly with environmental parameters are elliptic (on the left side) while modules that do not correlate with any environmental parameter are shown in rectangular form (on the right side). A positive correlation is shown by a straight line, a negative correlation by a dashed line. The width of the lines represents the strength of the correlations (see Supplementary Figure S6 for more information).

The network analysis revealed the presence of 15 interaction groups, or modules, of highly correlated bacterial and eukaryotic ASVs (Figure 8). Four of these modules comprised only Bacteria (Mod 5, 10, 13, and 14). In contrast, the remaining modules included both Bacteria and Eukarya. No modules that contain only Eukarya were found. Most modules that included both domains had more Bacteria than Eukarya, except for Mod 9, which had three times more Eukarya than Bacteria. The detailed taxonomic composition of the interaction groups is available in Supplementary Table S6.

The analysis of the bacterial composition of the modules showed that Actinobacteria was the dominant group, comprising 42.7% of all bacterial ASVs. Chloroflexi was the second most abundant group, accounting for 17.1% of the ASVs and dominating modules 6 and 7. Proteobacteria, Gemmatimonadetes, and Verrucomicrobia were present at a higher percentage of the ASVs, exceeding 5%. Meanwhile, the Acidobacteria and Patescibacteria represented less than 5% of the total ASVs. However, Acidobacteria was present in most modules, with at least one ASV in 10 modules. Some phyla, such as Firmicutes and Planctomycetes, were represented by only a single ASV.

Concerning the Eukarya, the analysis of the modules showed that all modules except for three modules (Mod 1, Mod 7, and Mod 12) contained fungi. Eight modules had green algae (Chlorophyta), mostly found in conjunction with fungi, potentially forming lichens in seven modules, and/or Cercozoa, likely feeding on algae in four modules. There was only one module (Mod 3) where fungi were the only eukaryotic group present. Cercozoa were never observed without the presence of Chlorophyta.

Each module was confined to specific sites of the glacier transect and sampling depths. The two ends of the transect, sites GT0 and GT80, had with four the highest number of modules per site. At GT0, the middle layer (Mod 3) and the deep layer (Mod 6) had unique eukaryotic compositions, with the only eukaryotes being the cold-adapted yeast-like basidiomycete *Glaciozyma*, naked amoeba *Flamella* (Amoebozoa) and Ascomycetes, respectively. Meanwhile, the surface module (Mod 4) at GT0 and all eight other modules with eukaryotes included green algae. At the GT65 site surface, the green algal ASVs were assembled with Chytridiomycetes, known for parasitizing green algae (Mod 15). At site GT80 surface, the streptophyte green alga *Klebsormidium*, Cercozoa, and lichen-forming Ascomycetes were associated with the bryophyte *Grimmia* (Mod 7).

Of the 15 modules, we found eight significantly correlated with environmental parameters at a significance level of *p* < 0.05. These modules included five ASVs that included Bacteria and Eukarya (Modules 1, 3, 7, 11, and 12) and three with only bacterial ASVs (Modules 5, 13, and 14). A closer examination of the correlations between each module and the various environmental parameters showed that distance from the glacier was related to Modules 3 and 5, while the type of soil minerals was linked to modules 11 and 12. In addition, pH, TC, and TN were found to be correlated with Module 7. A detailed description of these correlations between each module and the environmental parameters can be found in the Supplementary Figure S6.

## Discussion

The comparison of eDNA and iDNA pools identified three distinct modes reflecting differences in species abundances, microbial activity, and ecological conditions:

1. Similar abundances in both pools suggest a balanced turnover in the microbial community, indicating potentially viable microbial population maintaining ecosystem stability (Genderjahn et al., 2021; Bartholomäus et al., 2024). Unlike the Eukarya, where the distribution of taxonomic groups in both DNA pools was uneven, the similar abundance of bacteria in the eDNA and iDNA pools indicates metabolic adaptability to environmental conditions (Hogg et al., 2006; Levy-Booth et al., 2007). Up to 71% of bacterial ASVs were shared between eDNA and iDNA, increasing from GT0 to GT80 along the chronosequence suggesting bacterial persistence across environments.
2. Higher relative abundance of certain microbial taxa in the iDNA pool compared to the eDNA pool indicates potentially viable microbial growth and adaptation to the prevailing environmental conditions, such as *Methylocapsa* (Proteobacteria) and WPS-2. Also, bacteria-like *Pseudonocardia* ASV0005, Gemmatimonadaceae ASV0002, and Eremiobacterota ASV0001, along with the green algae *Chloroidium* ASV0004, *Desmococcus* ASV0008, and the bryophyte *Grimmia* ASV0001, thrive in nutrient-depleted conditions due to their carbon and bacterial nitrogen fixation capabilities (Housman et al., 2006).
3. Lower diversity and abundance in the iDNA pool relative to the eDNA pool, exemplified by Actinobacteria such as *Acidimicrobiia* and *Conexibacter*, may indicate constrained microbial communities in response to unfavorable environmental conditions, such as climate or nutrient availability and water availability.

The applied DNA separation method has already been used in various desert ecosystems (Schulze-Makuch et al., 2018, 2021; Genderjahn et al., 2021; Horstmann et al., 2024; Wang et al., 2024), which are also characterized by extremely dry and nutrient-poor conditions. Recently, Bartholomäus et al. (2024) demonstrated for the Atacama Desert that, despite potential limitations (e.g., dead but structurally intact cells), the iDNA pool almost exclusively represents living cells. Conversely, eDNA from dead organisms can persist for varying lengths of time—ranging from days to geological timescales—depending on factors such as pH and clay content (Ogram et al., 1988; Paget et al., 1992). Furthermore, caution is required when interpreting the differences between iDNA and eDNA, as the degradation of eDNA or its utilization as a nutrient source could contribute to a decrease in its abundance. In this study, we therefore consider iDNA as a marker for living and potentially viable microorganisms, while eDNA represents a spectrum ranging from recently deceased microbes to ancient microbial DNA.

In light of the modes and limitations discussed above, we observed the transition from pioneer to more complex microbial communities both horizontally along the deglaciation gradient and vertically with increasing soil depth, characterized by interactions between bacterial and eukaryotic taxa. While alpha diversity (iDNA) generally decreased with depth, the observed increase in microbial complexity and interactions in deeper soil layers may be driven by niche specialization, resource availability, and reduced disturbance. These factors can promote the development of coupled microbial community, even in environments with lower overall diversity. The distribution of taxonomic groups in both DNA pools was balanced for bacteria but uneven for eukaryotes, with the eDNA pool consistently larger across all study sites. The higher abundance of unique ASVs in the eDNA pool of eukaryotes suggests greater temporal variation compared to bacteria. This pattern may reflect transiently favorable environmental conditions driving succession patterns along the glacier transect (Eddie et al., 2010). While mobile multicellular eukaryotes shedding cells during dispersal could contribute to this variation, most of the detected eukaryotes were small, single-celled micro-eukaryotes, which may also be transported by transient liquid water or physical forces such as wind-driven sedimentation.

At pioneer-stage sites (GT0 and GT30), microbial communities appeared metabolically versatile, adapting to harsh conditions characterized by low nutrient content, high salinity, and high pH values (Bajerski and Wagner, 2013). Nitrogen-fixing representatives of Cyanobacteria, Chloroflexi, and Proteobacteria were abundant, likely channeling nitrogen to other microorganisms. For example, genera such as *Methylocapsa* were detected, which are known for their metabolic adaptability, including nitrogen fixation. Despite limited water availability, green algae (e.g., *Chloroidium*, *Deuterostichococcus*, *Neocystis*, *Prasiola*) act as pioneering primary producers. These algal genera are well known from soils and other dry terrestrial habitats and have also been identified in fellfield soils of ice-free Maritime Antarctica (Darienko and Friedl, 2021; Rybalka et al., 2023). As photoautotrophs, they appear particularly adapted to dry, nutrient-depleted environments. In contrast, other green algae, such as *Raphidonema* and *Chloromonas*, were only recovered from the site closest to the glacier front. These taxa are known to be associated with the phenomenon of “colored snow” on snowfields and glaciers in high mountain and polar regions (Matsuzaki et al., 2021). Their presence may be the result of introduction via ephemeral meltwater from glacier ice. Other heterotrophic eukaryotes, including *Glaciozyma* and non-lichen-forming ascomycetes, likely utilize organic compounds provided by bacteria and green algae (Niederberger et al., 2015; Wei et al., 2015; Ji et al., 2017).

Interestingly, these pioneer sites are notably enriched with pioneering green algae, particularly members of the Trebouxiophyceae and Chlorophyceae. These photoautotrophic eukaryotes appear to be among the first colonizers in the early stages of soil formation following deglaciation. In addition, several abundant microbial phyla, including Chloroflexi, Gemmatimonadetes, and Proteobacteria, which are known to thrive in extreme environments (Schulze-Makuch et al., 2018) and oligotrophic niches (Ranjani et al., 2016; Rime et al., 2016; Wei et al., 2016), play a significant role in initial soil development (Zhang et al., 2016) at these sites. In contrast, the phylum Actinobacteria, also known for its role in early colonization and metabolite production (Babalola et al., 2009; Ganzert et al., 2011; Fernández-Martínez et al., 2017; Jiang et al., 2018; Garrido-Benavent et al., 2020), was highly abundant across all sites.

This early colonization is further supported by the presence of cryophilic fungi, such as *Leucosporidium* and *Glaciozyma*, which appear to thrive by utilizing organic matter produced by both bacteria and green algae. Notably, this pattern of colonization was consistent across different soil depths, from the surface to deeper layers (~20 cm), suggesting robust interactions between photoautotrophs and heterotrophic microorganisms during the initial stages of ecological succession. While it remains uncertain whether this represents a general trend across various glacier forefields or a site-specific phenomenon, our findings suggest a new working hypothesis: the simultaneous colonization by bacteria and photoautotrophic eukaryotes plays a critical role in the early establishment of microbial communities in recently deglaciated environments. Future studies in other glacier forefields are required to assess the broader applicability of this ecological pattern.

At the middle sites, GT55 and GT60, environmental conditions likely promoted microbial diversity and interactions. This is evidenced by lichen symbioses involving Pezizomycotina (Ascomycetes) and green algae (Trebouxiophyceae), as well as putative parasitic interactions between Chytridiomycetes and green algae (Figure 6; Supplementary Figure S3). High silt content, elevated conductivity, and low sand content likely influenced microbial community composition, with Acidobacteria thriving in silty niches. The surface layer harbored a greater number of ASVs, possibly because silt particles provide a favorable environment for bacterial attachment and nutrient acquisition. Additionally, the presence of *Methylocapsa* at mid-chronosequence sites (GT55, GT65) suggests that this taxon colonized the soil and facilitated the aerobic oxidation of atmospheric methane (Li et al., 2014; Hegyi et al., 2021).

Farther from the glacier (GT80), reduced water availability, coupled with higher carbon content and acidic soil properties, led to a zonation pattern shaping microbial community structure (Eddie et al., 2010). The streptophyte green alga *Klebsormidium* and the bryophyte *Grimmia* were found exclusively at this site, alongside algae-feeding Cercozoa and lichen-forming Ascomycetes. *Pseudonocardia*, known for its autotrophic growth, co-occurred with *Grimmia*, possibly benefiting from H₂ production during organic matter decomposition (Park et al., 2008; Grostern and Alvarez-Cohen, 2013).

Phototrophic cyanobacteria, although generally low in abundance and sparsely distributed along glacier forefields, play a key role by fixing carbon and nitrogen to support other microbes (Musat et al., 2008; Pester et al., 2010). Their low abundance likely reflects their concentration in topsoil layers, optimizing light exposure and moisture access (Ji et al., 2016; Garrido-Benavent et al., 2020). Notably, Leptolyngbyaceae were detected throughout the chronosequence, potentially contributing to early colonization (Turicchia et al., 2005; Frey et al., 2013). The co-occurrence of Nostocales cyanobacteria with *Grimmia* at site GT80 suggests a potential mutualistic relationship (Davey, 1983; Ino and Nakatsubo, 1986).

Similar to cyanobacteria, *Methylocapsa* exhibited uneven abundance, peaking in mid-chronosequence sites (GT55, GT65; Figure 5). This genus is known for nitrogen fixation (Auman et al., 2001; Liebner and Svenning, 2013) and is crucial in nutrient-limited environments (Vile et al., 2014), as well as for methane oxidation in cold soils (Bárcena et al., 2011). Acidimicrobiia, Gemmatimonadaceae, and Saccharimonadales, likely favored by initial high salinity near the glacier (GT0, GT30), were absent at the mature site (GT80).

Vertical distribution within soil profiles also reflected habitat preferences. For example, methane-oxidizing *Methylocapsa* (ASV_0009, ASV_0026) increased in abundance with depth at the mature site (GT80), potentially due to a pH and nutrient gradient created by the bryophyte cover. Similar patterns were observed in other sites (GT55, GT65; Figure 5). This depth-dependent distribution aligns with previous findings in this chronosequence (Bajerski and Wagner, 2013) and other glacier forefield studies (Sigler et al., 2002; Noll and Wellinger, 2008; Zhao et al., 2021), suggesting deeper layers in mature sites resemble less-developed surface soils.

Green algae, particularly Trebouxiophyceae, dominated the photoautotrophic eukaryotes (Figure 6). They exhibited a clear succession pattern along the chronosequence, with the highest diversity observed near the glacier. This aligns with their known role as early colonizers in harsh environments (Rybalka et al., 2023). Interestingly, Trebouxiophyceae DNA (e.g., *Chloroidium* sp., *Desmococcus* sp.) and *Neocystis* sp. (Chlorophyceae) persisted even in deeper soil layers lacking light penetration. This persistence might be due to potential heterotrophic capabilities reported for desert green algae *Chloroidium* (Nelson et al., 2017) or physical movement by cryoturbation or wind (Broady, 1981). In contrast, Ulvophyceae and Xanthophyceae algae were predominantly detected in the eDNA pool. Although this suggests a historical role in colonization, their absence in the iDNA pool might be due to challenges in DNA extraction, particularly for delicate eukaryotic cells such as these algae (Rybalka et al., 2009; Škaloud and Rindi, 2013). These algae groups are nonetheless established pioneers in diverse extreme environments, as reported in Alpine glacier forefields (Frey et al., 2013), Himalayan barren soils (Schmidt et al., 2011; Janatková et al., 2013), and Antarctic fellfield soils (Rybalka et al., 2023).

Unlike algae, the bryophyte *Grimmia* was abundant only at the mature site (GT80). Its presence might create a nutrient oasis, influencing the microbial community below through a vertical nutrient gradient (Barrett et al., 2006). This could explain the shift toward a more heterotrophic, macromolecule-degrading community observed at other locations in Antarctica (Ganzert et al., 2011). However, the patchy distribution of *Grimmia* limits its impact to localized areas. Geochemical factors like pH and nutrient availability likely significantly shape the overall microbial community structure across the glacier transect (Barrett et al., 2006).

The Basidiomycete *Glaciozyma* and some ascomycetes (*Alatospora*, *Lecanicillium*, *Neofabraea*, and *Simplicillium*) were the most abundant fungi (Figure 6). These groups are reported in various Antarctic habitats (Romeike et al., 2002; Fernández-Martínez et al., 2017; Garrido-Benavent et al., 2020; Santos, Dos Santos et al., 2020), including permanently ice-covered lakes (Rojas-Jimenez et al., 2017), McMurdo Dry Valleys soils (Thompson et al., 2020), and even Antarctic permafrost rock glaciers (Sannino et al., 2023), and likely play key roles in organic matter decomposition and nutrient cycling (Tedersoo et al., 2014; Treseder and Lennon, 2015).

The DNA separation method allowed the application of Indicator Species Analysis (ISA) to the iDNA pool, excluding eDNA, to identify distinct living bacterial ASVs as either specialists and generalists across the transect. Our findings highlight the prevalence of specialist bacterial ASVs, particularly within the phyla Actinobacteria and Chloroflexi, which were primarily associated with specific successional stages. The identification of these specialists is based on their restricted distribution across the transect, indicating their adaptation to specific environmental conditions present at each site. Specialists, by thriving in the prevailing soil conditions, likely play a crucial role in stabilizing the newly exposed soil and contributing to ecosystem development at these specific locations (Fierer and Jackson, 2006; Bardgett and van der Putten, 2014; Delgado-Baquerizo et al., 2016). In contrast, only a few bacterial ASVs, such as those belonging to Actinobacteria (e.g., ASV 0035) and certain eukaryotic ASVs (phototrophic green algae, Trebouxiophyceae), exhibited generalist tendencies. These generalists were observed to have a broad distribution across multiple sites, suggesting their ability to adapt to varying environmental conditions (e.g., *Deuterostichococcus*, *Elliptochloris* with lichen symbioses; Beck et al., 2019), including those associated with glacial retreat. The predominance of specialists at sites farther from the glacier (e.g., GT80) highlights the importance of site-specific environmental factors in shaping microbial community composition. These specialists may contribute to soil stability and nutrient cycling in these unique habitats. In contrast, the limited number of generalists across the transect suggests that the microbial communities are largely driven by localized environmental conditions.

Network analysis revealed distinct co-occurrence patterns for bacteria and eukaryotes. Co-existence within modules likely reflects shared niches, metabolic dependencies, or interactions like parasitism/symbiosis (Barberán et al., 2012; Zelezniak et al., 2015; Vimercati et al., 2022). Notably, bacterial communities displayed a more deterministic assembly driven by environmental factors such as pH, nutrients, temperature, and UV (Jiang et al., 2018; Tripathi et al., 2018; Vimercati et al., 2022). Conversely, less-connected eukaryotic networks suggest a greater influence of chance events (dispersal limitation, ecological drift) on their assembly (Stegen et al., 2013). While our analysis did not explicitly apply the framework proposed by Stegen et al. (2013), the observed patterns align with their principles and highlight the potential role of stochastic processes in shaping eukaryotic communities. Future studies could benefit from a more formal application of this framework to further elucidate the mechanisms driving community assembly in these environments. The sites closest to (GT0-GT30) and farthest (GT80) from the glacier exhibited the most complex interactions, with a dip in the middle of the transect (GT55-GT65), a pattern also observed in other studies (Vimercati et al., 2022). Environmental factors likely drove community shifts due to the barren soils. However, niche partitioning due to environmental factors might have played a stronger role in shaping early-stage communities. This is further supported by weaker correlations between community composition and environmental factors at this site. The complex network at GT80 suggests potential vulnerability to disturbances due to interdependent relationships. The late-stage community appeared more stable, but its complex network suggests vulnerability to the loss of key taxa due to tight interdependencies. However, co-occurrence patterns do not necessarily imply direct interactions. Shared responses to environmental factors or similar functions can also contribute.

## Conclusion

Our study applied DNA separation techniques to uncover living microbial communities in Antarctic ice-free oases. By distinguishing between iDNA and eDNA, we gained insights into microbial succession and community assembly across a glacier forefield chronosequence. We identified three distinct modes of microbial distribution, reflecting adaptability, and persistence under changing environmental conditions. In early successional stages, pioneering green algae (Trebouxiophyceae, Chlorophyceae) and bacteria co-colonized glacier forefields, accompanied by cryophilic fungi – a key interaction that may drive initial soil formation post-deglaciation. As succession progressed, bacterial communities were shaped by deterministic processes, while stochastic factors played a greater role in eukaryotic community assembly. Our findings also highlight potential symbiotic relationships between prokaryotic and eukaryotic microorganisms, contributing to complex Antarctic soil ecosystems. The persistence of eDNA suggests past extinction events among bacterial groups, likely due to environmental shifts, reinforcing the essential role of prokaryotes in early soil development. Overall, this research enhances our understanding of microbial ecology in polar regions and underscores the need to refine DNA-based methods to unravel the mechanisms driving community assembly in extreme, low-biomass ecosystems.

## Data Availability

The datasets presented in this study can be found in online repositories. The names of the repository/repositories and accession number(s) can be found in the article/Supplementary material.

## Footnotes

^1^ http://www.venndiagrams.net
