# Supplementary material for: From single pioneers to complex pro- and eukaryotic microbial networks in soils along a glacier forefield chronosequence in continental Antarctica
Source: Front Microbiol. 2025 May 21;16:1576898. doi: 10.3389/fmicb.2025.1576898 (PMC12133861; doi:10.3389/fmicb.2025.1576898)
Supplement: Supplementary file 1 [file Supplementary_file_1.zip › Table S5.DOCX]

**Table S5.** Network topology factors for microbial networks across the soil chronosequence including nodes, number of correlations between nodes (edges), average number of connections per node in the network (average degree), tendency of nodes to form clusters or groups (clustering coefficient), and average distance between all pair of nodes in the network (average path length).

| Topology factor | Microbial communities | Bacterial communities | | | | | | Eukaryotic communities | | | | | |
| --- | --- | --- | --- | --- | --- | --- | --- | --- | --- | --- | --- | --- | --- |
|  | Site | GT0 | GT30 | GT55 | GT65 | | GT80 | GT0 | GT30 | | GT55 | GT65 | GT80 |
| Number of edges | 7278 | 1572 | 400 | 644 | 835 | | 1256 | 119 | 87 | | 67 | 78 | 111 |
|  |  | Bac. – Bac. 4777 | | | | Bac. – Euk. 2039 | | | | Euk. – Euk. 462 | | | |
| Nodes | 505 | 109 | 49 | 64 | 81 | | 100 | 22 | 20 | | 19 | 14 | 27 |
|  |  | 403 | | | | | | 102 | | | | | |
| Average degree | 14.41 | 14.42 | 8.16 | 10.06 | 10.31 | | 12.56 | 5.41 | 4.35 | | 3.52 | 5.57 | 4.11 |
| Modularity^*^ | 0.83 | 0.80 | | | | | | 0.84 | | | | | |
| Clustering coefficient | 0.82 | 0.79 | | | | | | 0.97 | | | | | |
| Average path length | 5.1 | 5.0 | | | | | | 1.5 | | | | | |

^*^Network with modularity value > 0.4 indicates that nodes are densely connected within their own module, but sparsely connected to nodes in other modules.
