# Supplementary material for: From single pioneers to complex pro- and eukaryotic microbial networks in soils along a glacier forefield chronosequence in continental Antarctica
Source: Front Microbiol. 2025 May 21;16:1576898. doi: 10.3389/fmicb.2025.1576898 (PMC12133861; doi:10.3389/fmicb.2025.1576898)
Supplement: Supplementary file 1 [file Supplementary_file_1.zip › Supplementary Figures.DOCX]

Supplementary Material

# Supplementary Data

The 16S and 18S rRNA gene amplicon sequences reported in this paper have been deposited in the NCBI database (accession no. PRJNA68595 and ID 685954).

# Supplementary Figures and Tables

## Supplementary Figures


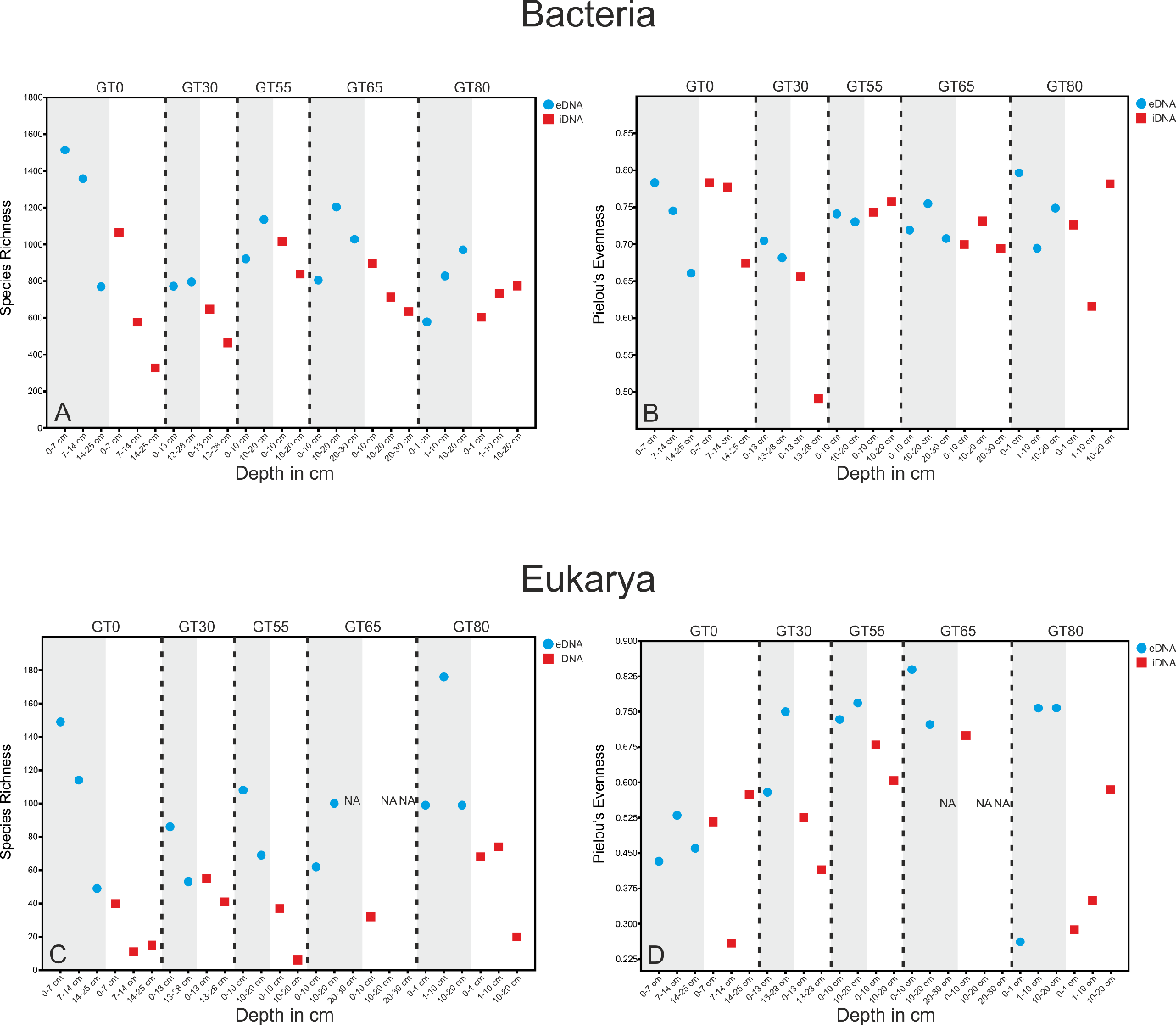


**Supplementary Figure S1.** Plot showing the Species Richness (left panel) and Pielou’s Evenness (right panel) for Bacteria (A, B) and Eukarya (C, D) along the soil chronosequence (sites GT0 to GT80, closest to farthest from the glacier tongue) and different depths for each site. The eDNA pool is characterized by a grey background and blue circles. The iDNA pool is characterized by a white background and red squares. Data points represent mean values of duplicate samples.


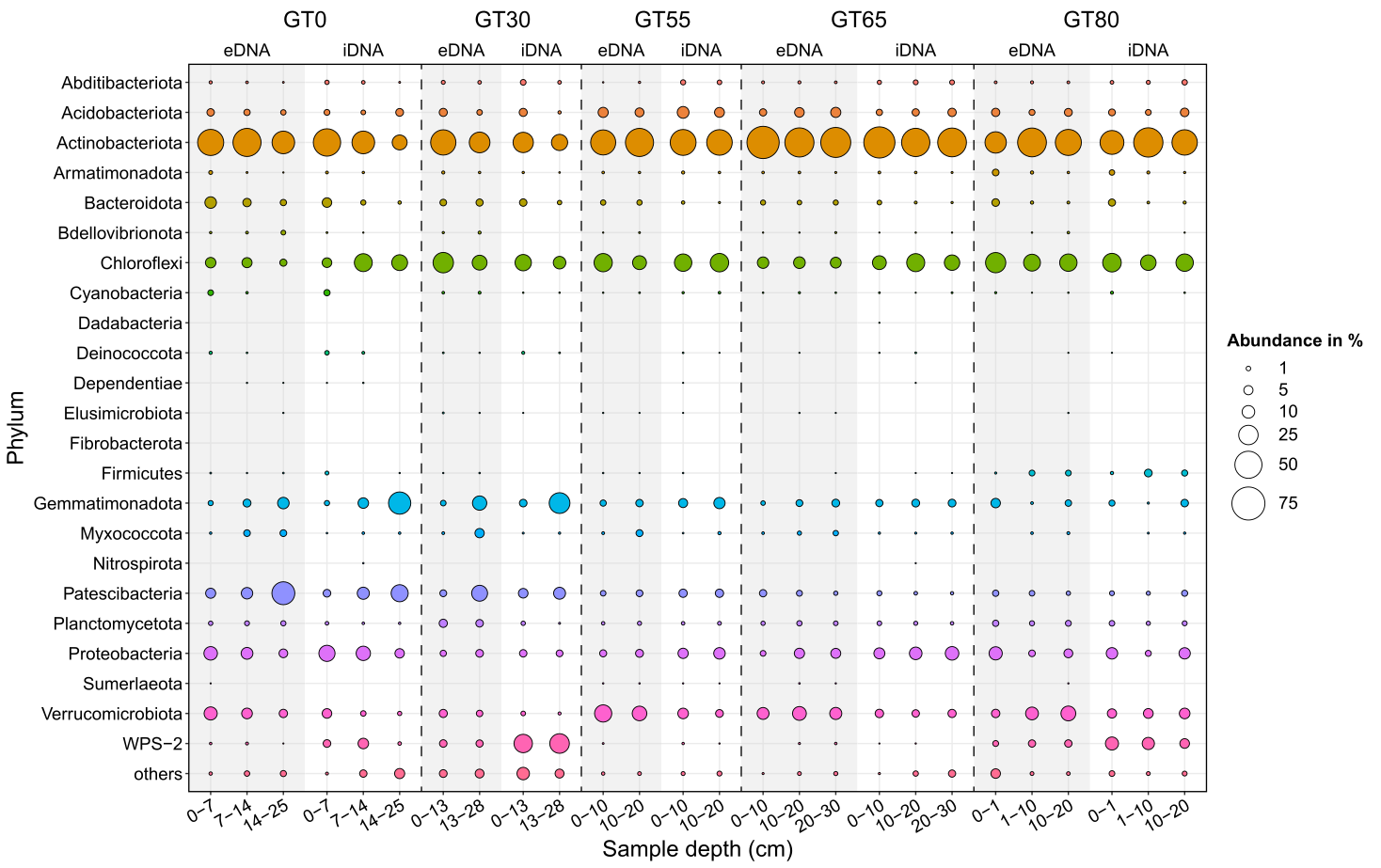


**Supplementary Figure S2.** Bubble plot showing the distribution and community structure of Bacteria at phylum level for both separated DNA pools (eDNA, grey background; iDNA, white background) along the transect. Each filled circle represents the occurrence of a certain phylogenetic group with the size corresponding to their relative abundance.


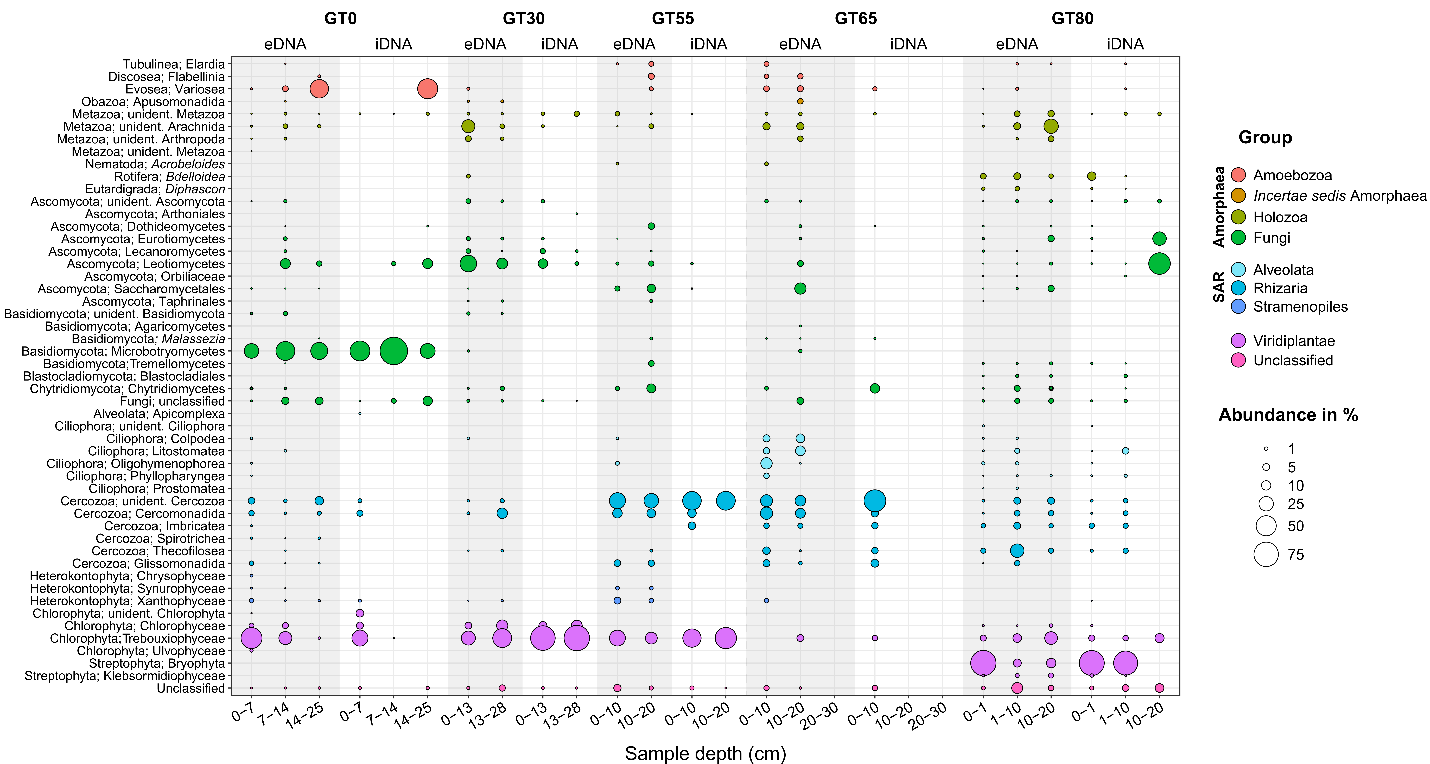


**Supplementary Figure S3.** Bubble plot showing the community structure and distribution of Eukarya at higher taxa level in both separated DNA pools (eDNA, grey background; iDNA, white background). Each filled circle represents the occurrence of a certain phylogenetic group with the size corresponding to their relative abundance. NA - no data available.


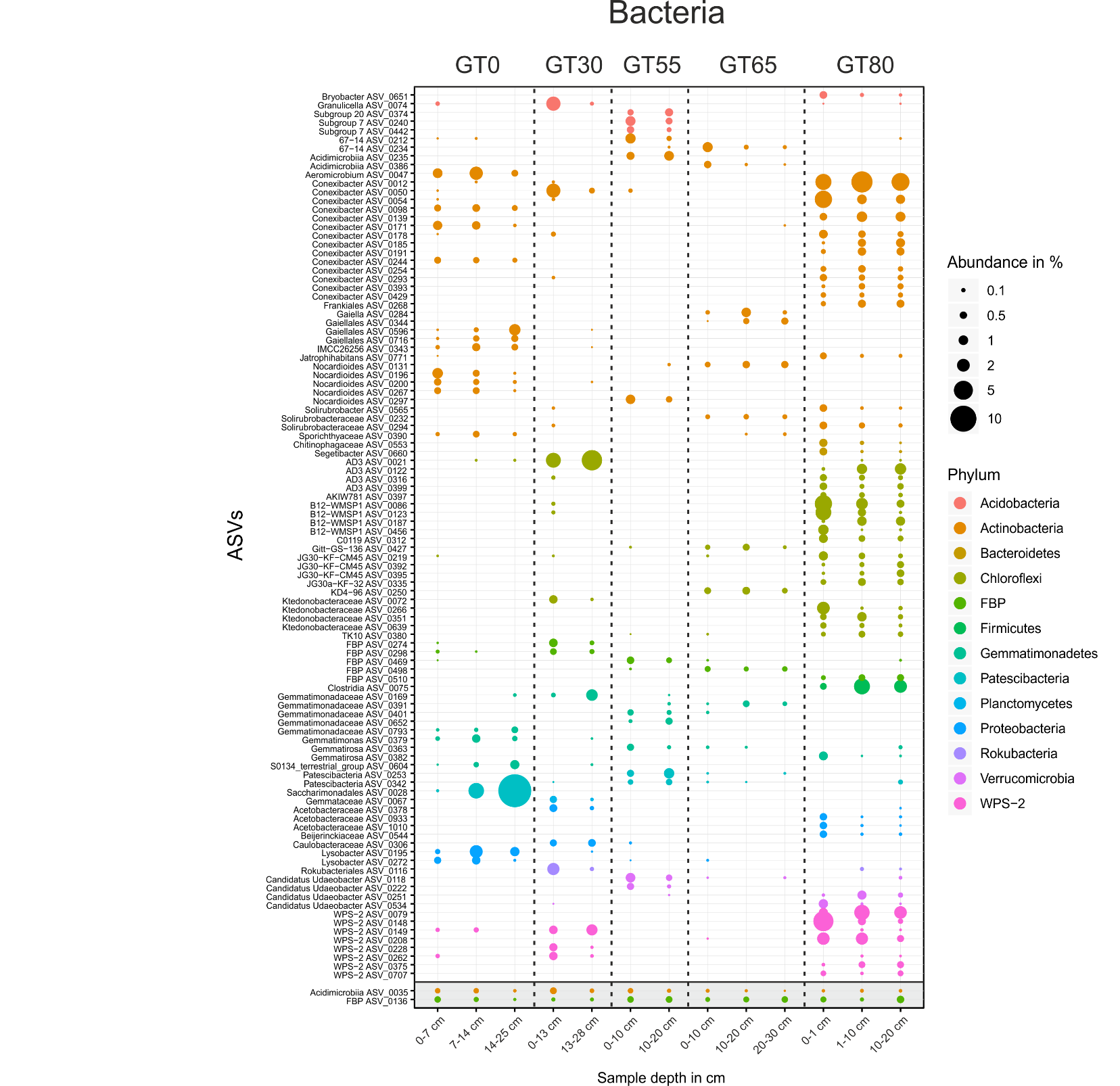


**Supplementary Figure S4.** Bubble plot showing bacterial specialist (white background) and generalist ASVs (grey background) of the iDNA pool, based on an indicator species analysis (ISA).


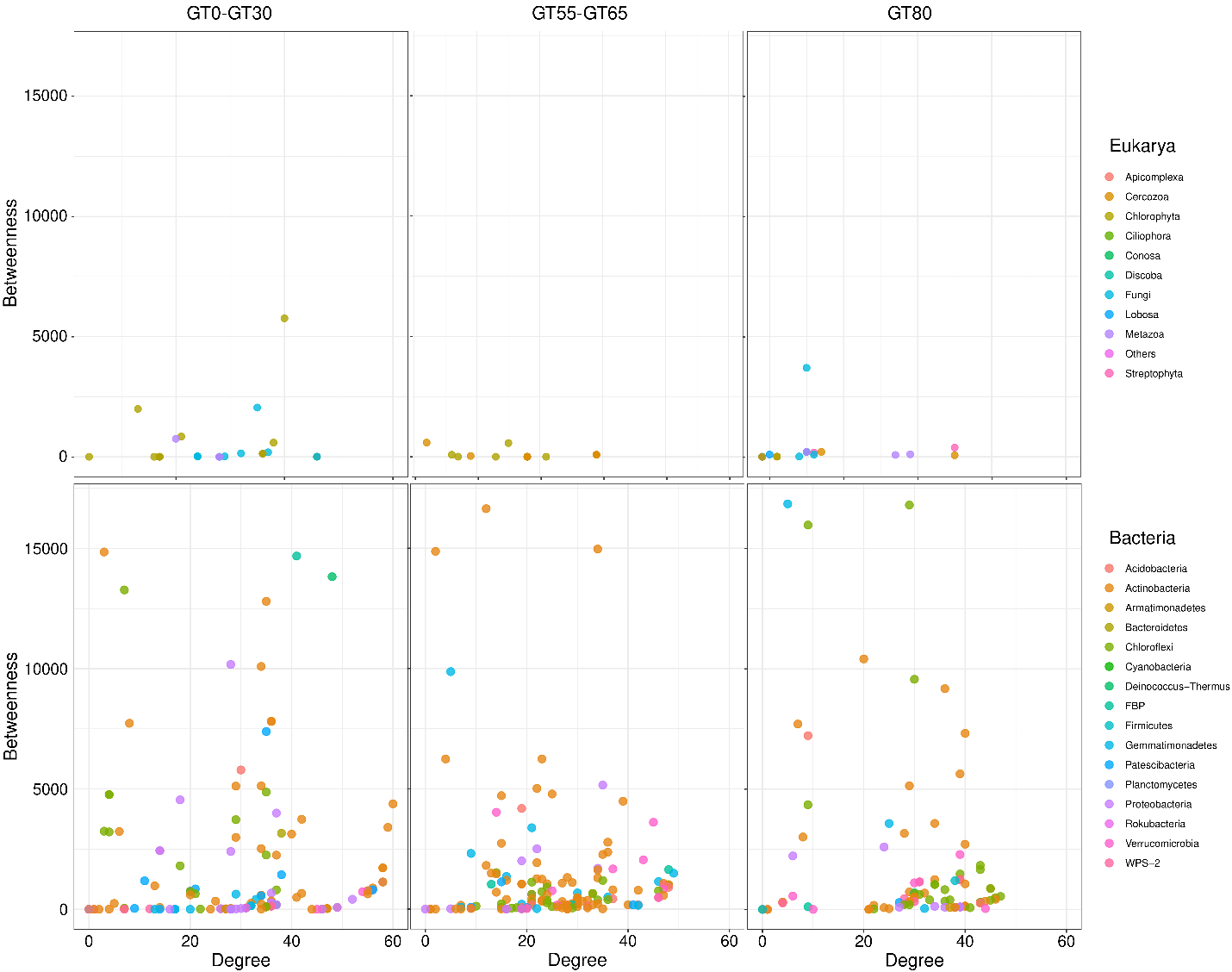


**Supplementary Figure S5.** Betweenness centrality and degree of each ASV in the networks. Keystone taxa are the nodes with high betweenness centrality and high degree values.


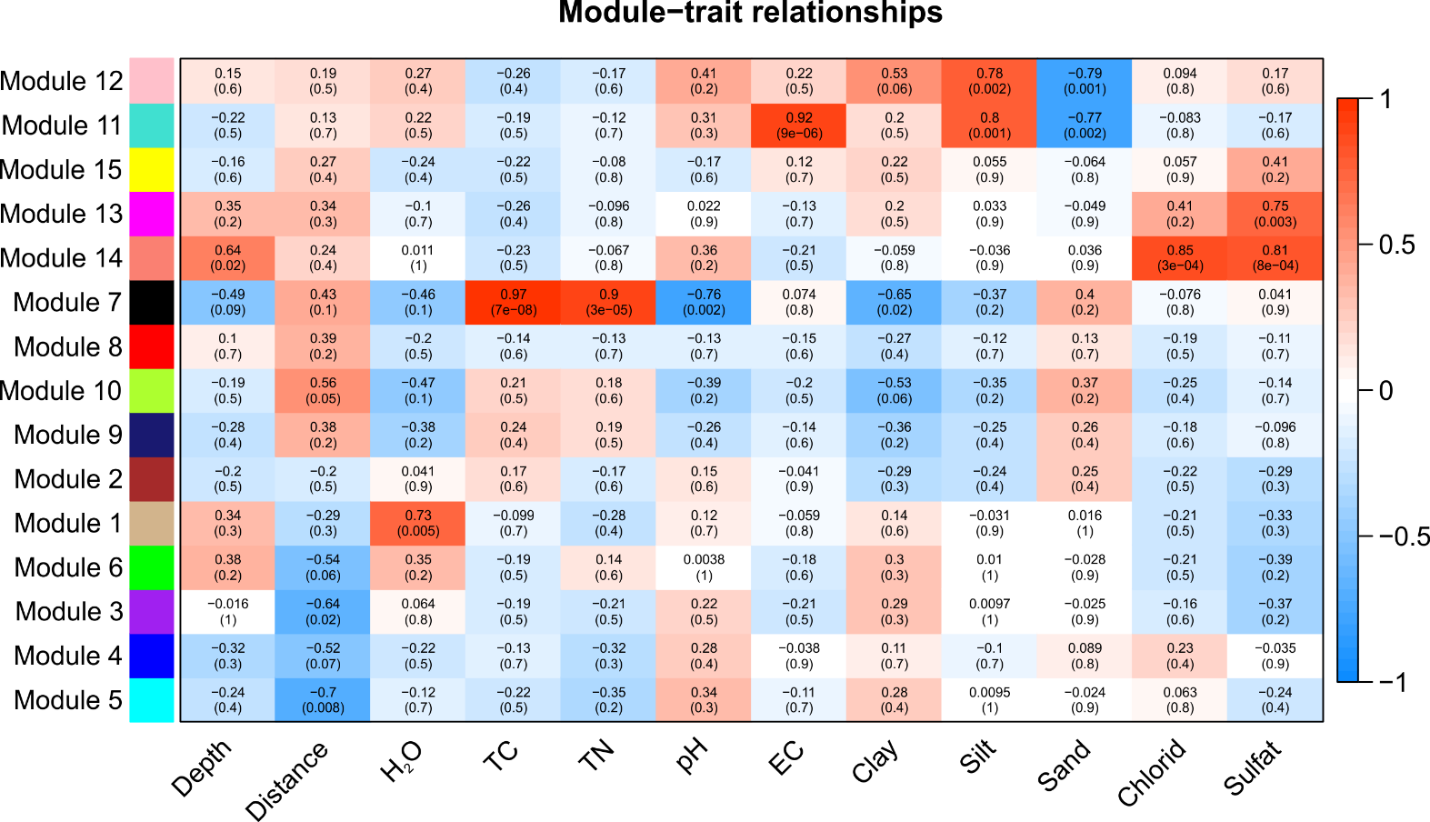


**Supplementary Figure S6.** Module-trait relationships showing the correlation of each module with a specific environmental parameter. The color scale represents the strength of the correlation, with red displaying a positive correlation and blue displaying a negative correlation, respectively. The values in each box represent the correlation value (upper) followed by the p-value (in parentheses, lower). TC – total carbon, TN – total nitrogen, EC – electrical conductivity.

## Supplementary Tables

All other data are available in Tables S1-S6.
