# Supplementary material for: From single pioneers to complex pro- and eukaryotic microbial networks in soils along a glacier forefield chronosequence in continental Antarctica
Source: Front Microbiol. 2025 May 21;16:1576898. doi: 10.3389/fmicb.2025.1576898 (PMC12133861; doi:10.3389/fmicb.2025.1576898)
Supplement: Supplementary file 1 [file Supplementary_file_1.zip › Table S1.DOCX]

**Overview of the samples and information on texture and structure of the soil**

Table S1 Information on the locations of the samples and the soil structure at these locations. GT – Glacier Transect, numbers specify the distance from the glacier in meter.

| Site | Distance (m) | depth (cm) | Coordinates | Description |
| --- | --- | --- | --- | --- |
| GT80 | 80 | 0-1 | S 69° 24,135; E 76° 20,296 | Surface covered in moss |
|  | 80 | 1-10 |  | Stones |
|  | 80 | 10-20 |  | Solid rock |
| GT65 | 65 | 0-10 | S 69° 24,137; E 76° 20,273 | moist |
|  | 65 | 10-20 |  | moist |
|  | 65 | 20-30 |  | Stones |
| GT55 | 55 | 0-10 | S 69° 24,137; E 76° 20,258 | Stones |
|  | 55 | 10-20 |  | frozen, dry; Stones |
| GT30 | 30 | 0-13 | S 69° 24,139; E 76° 20,222 | Stones |
|  | 30 | 13-28 |  | frozen, dry; Stones |
| GT0 | 0 | 0-7 | S 69° 24,140; E 76° 20,178 | Dry |
|  | 0 | 7-14 |  | Moist |
|  | 0 | 14-25 |  | Permafrost |
