# Supplementary material for: From single pioneers to complex pro- and eukaryotic microbial networks in soils along a glacier forefield chronosequence in continental Antarctica
Source: Front Microbiol. 2025 May 21;16:1576898. doi: 10.3389/fmicb.2025.1576898 (PMC12133861; doi:10.3389/fmicb.2025.1576898)
Supplement: Supplementary file 1 [file Supplementary_file_1.zip › Table S2.DOCX]

**Soil Properties along a Glacier Forefield Chronosequence in Continental Antarctica (Larsemann Hills, East Antarctica)**

Table S2: Selected soil properties in the glacier forefield Larsemann Hills, East Antarctica. GT – Glacier Transect, numbers specify the distance from the glacier in meters. Values represent the z-score to standardize data for comparison, except for H_2_O, C, and N, which are given in percentage.

| Site | depth (cm) | % H_2_O | C (%) | N (%) | pH | EC | Clay | Silt | Sand | Chlorid mg/l | Sulfat mg/l |
| --- | --- | --- | --- | --- | --- | --- | --- | --- | --- | --- | --- |
| GT80 | 0-1 | 0.238 | 0.397 | 0.121 | -2.357 | 0.478 | -1.720 | -0.900 | 0.978 | -0.020 | 0.3152 |
|  | 1-10 | 0.281 | 0.112 | 0.096 | -0.665 | -0.501 | -1.007 | -0.766 | 0.794 | -0.560 | -0.3212 |
|  | 10-20 | 0.789 | 0.000 | 0.089 | -0.516 | -0.659 | -0.914 | -0.610 | 0.641 | -0.547 | -0.3833 |
| GT65 | 0-10 | 0.505 | 0.000 | 0.091 | -0.832 | 0.134 | 0.474 | -0.293 | 0.258 | -0.129 | 0.9207 |
|  | 10-20 | 1.166 | 0.000 | 0.091 | -0.627 | -0.501 | 0.765 | -0.053 | -0.017 | -0.101 | 1.1380 |
|  | 20-30 | 1.222 | 0.000 | 0.091 | 1.233 | -0.464 | -1.033 | -0.656 | 0.700 | 3.033 | 2.3799 |
| GT55 | 0-10 | 2.094 | 0.000 | 0.090 | 0.870 | 3.154 | 0.315 | 2.314 | -2.201 | -0.285 | -0.7248 |
|  | 10-20 | 2.201 | 0.000 | 0.089 | 1.019 | -0.307 | 1.637 | 1.900 | -1.951 | -0.029 | 0.1755 |
| GT30 | 0-13 | 1.403 | 0.111 | 0.088 | 0.470 | -0.165 | -0.954 | -0.786 | 0.815 | -0.592 | -0.7404 |
|  | 13-28 | 3.686 | 0.000 | 0.084 | 0.265 | -0.083 | 0.580 | 0.091 | -0.144 | -0.436 | -0.6938 |
| GT0 | 0-7 | 0.613 | 0.000 | 0.083 | 0.833 | -0.075 | 0.315 | -0.308 | 0.276 | 0.886 | 0.0513 |
|  | 7-14 | 1.468 | 0.000 | 0.087 | 0.544 | -0.561 | 0.778 | 0.151 | -0.185 | -0.551 | -0.9732 |
|  | 14-25 | 2.352 | 0.000 | 0.099 | -0.237 | -0.449 | 0.765 | -0.085 | 0.035 | -0.669 | -1.1440 |
